# Prussian Blue Analogue-Templated Nanocomposites for Alkali-Ion Batteries: Progress and Perspective

**DOI:** 10.1007/s40820-024-01517-y

**Published:** 2024-09-26

**Authors:** Jian-En Zhou, Yilin Li, Xiaoming Lin, Jiaye Ye

**Affiliations:** 1 Key Laboratory of Theoretical Chemistry of Environment, Ministry of Education, School of Chemistry, South China Normal University, Guangzhou, 510006 People’s Republic of China; 2 School of Chemistry and Physics, Faculty of Science, Queensland University of Technology, 2 George Street, Brisbane, QLD 4000 Australia; 3 Centre for Materials Science, Queensland University of Technology, 2 George Street, Brisbane, QLD 4000 Australia

**Keywords:** Prussian blue analogues, Self-sacrificial template, Lithium-ion batteries, Sodium-ion batteries, Potassium-ion batteries

## Abstract

Lithium-ion batteries (LIBs) have dominated the portable electronic and electrochemical energy markets since their commercialisation, whose high cost and lithium scarcity have prompted the development of other alkali-ion batteries (AIBs) including sodium-ion batteries (SIBs) and potassium-ion batteries (PIBs). Owing to larger ion sizes of Na^+^ and K^+^ compared with Li^+^, nanocomposites with excellent crystallinity orientation and well-developed porosity show unprecedented potential for advanced lithium/sodium/potassium storage. With enticing open rigid framework structures, Prussian blue analogues (PBAs) remain promising self-sacrificial templates for the preparation of various nanocomposites, whose appeal originates from the well-retained porous structures and exceptional electrochemical activities after thermal decomposition. This review focuses on the recent progress of PBA-derived nanocomposites from their fabrication, lithium/sodium/potassium storage mechanism, and applications in AIBs (LIBs, SIBs, and PIBs). To distinguish various PBA derivatives, the working mechanism and applications of PBA-templated metal oxides, metal chalcogenides, metal phosphides, and other nanocomposites are systematically evaluated, facilitating the establishment of a structure–activity correlation for these materials. Based on the fruitful achievements of PBA-derived nanocomposites, perspectives for their future development are envisioned, aiming to narrow down the gap between laboratory study and industrial reality.

## Highlights

- The synthetic protocols of various Prussian blue analogue (PBA)-templated nanocomposites are discussed.
- Alkali-ion storage mechanisms based on intercalation, alloying, or conversion reactions are analysed.
- The properties of PBA-templated nanocomposites in alkali-ion batteries (AIBs) are evaluated and compared to outline the structure–activity correlation.
- Perspectives for the future development of PBA-templated AIB electrodes are envisaged.

## Graphical abstract

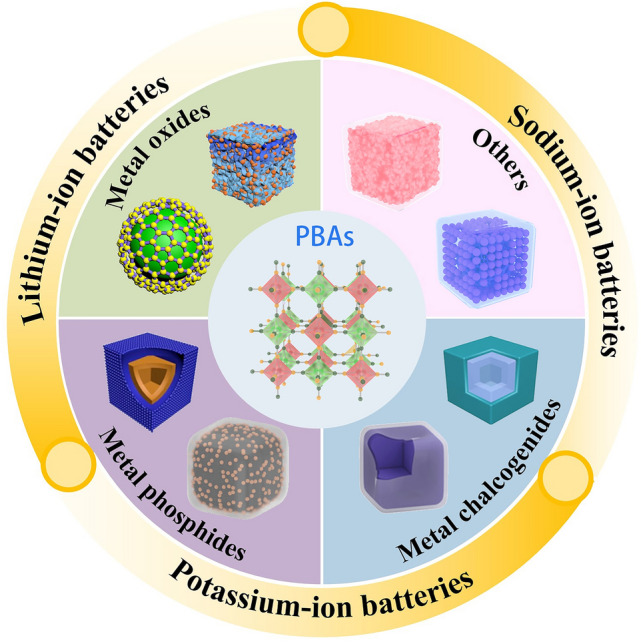

## Introduction

Renewable energy resources such as wind, solar, and tide have garnered increasing attention due to the depletion of fossil fuels, whose intermittence has driven the quest for efficient energy storage systems. Among these, as indispensable members of alkali-ion batteries (AIBs), lithium-ion batteries (LIBs) have occupied a predominant place in human life powering portable electronic devices and electrical vehicles (EVs) since their commercialisation in 1991 by Sony [1–4]. With the continued growth of the LIBs market, higher demands are raised, including lower cost, higher energy density, resource preservation, and greater safety. To address the skyrocketing price and scarcity of Li (~ 12,000 USD per tonne in May 2024, battery grade lithium metal), a significant interest in “beyond Li” batteries has been triggered, where sodium-ion batteries (SIBs) and potassium-ion batteries (PIBs) are viable alternatives for LIBs due to their abundant natural resources, cost-effectiveness, and similar working mechanisms [5]. For these AIBs, the kinetic and capacity mismatch between cathodes and anodes severely hampers their advancement [6–8], which urgently requires further design and optimisation of electrode materials.

To controllably fabricate AIB electrode materials with specific compositions and morphologies, it is viable to employ self-sacrificial templates. Metal–organic frameworks (MOFs), a typical type of porous crystalline organic–inorganic hybrid materials, have been intensively investigated as templates/precursors for energy materials with well-developed porosity, tailorable chemical composition, and desirable functionality [9–13]. Consisting of secondary building units (metal entities and bridging ligands), MOFs with structural periodicity and open pores are versatile templates to guarantee phase uniformity and large surface area of targeted products. As a symbolic family of MOFs, Prussian blue analogues (PBAs) with the open framework structure comprising metal cations bridged by cyanide groups can exert advantages including abundant diffusion channel for charge carrier ions, adjustable metal nodes, structural rigidity, and easy preparation when applied in the energy arena [14]. The ordered structure with interstitial spaces and metal centres enables the Faradic intercalation reaction and ion/mass transportation. Considering the redox activities provided by metal centres, primitive PBAs have been studied as lithium/sodium/potassium storage materials, during which phase transformation may occur with the insertion/extraction of alkali ions [15]. Nevertheless, there is still a long way to the practical use of pristine PBA electrodes for AIBs due to their intrinsic drawbacks including irreversible phase distortion upon cycling, structural vulnerability induced by crystal defects and coordinated water, sluggish ionic/electronic conductivity, and severe side reactions [14]. Although innumerable efforts have been devoted to mitigating the structural distortion related to multiphase behaviours by optimisation of crystal configuration, morphology, and composition [16, 17], the specific capacity and rate capability of PBAs lag far behind the industrial requirement of AIBs (particularly in full cells).

To evade the above drawbacks of primitive PBAs in alkali-ion storage and simultaneously utilise their structural advantages, PBAs have gained specific research interests as self-sacrificial templates. During the PBA-templated synthetic route, one factor predisposing PBAs to be ideal templates is their flexible and facile synthesis, of which the coprecipitation method remains the most dominant approach [18]. This coprecipitation synthetic method inevitably brings interstitial water leading to occupation of host ion insertion sites and aggravation of the structural dissolution [19], for which some methods such as surface modification [20], template method [21], single ion resource method [22], and adjustment of processing conditions (e.g. temperature and solvent environment) [23] have been proposed to optimise the PBA structure and composition. The PBA-templated method has been employed for the directional fabrication of nanocomposites with hierarchical porosity [24]. This synthetic route can not only retain the open framework structure with transport pathways for alkali ions but also enable the even distribution of active components with exceptional electrochemical activities. A range of active nanomaterials (porous carbons, metal oxides, metal carbides, metal chalcogenides, metal phosphides, metal hydroxides, etc.) are accessible via in situ thermal conversion or solution-based reactions with PBAs as self-sacrificial templates [15]. The prime advantages of adopting PBAs and their composites as precursors/templates are as follows: (i) the formed hollow architectures (i.e. core–shell, hollow shell, multi-shell) with controllable geometries and particle sizes allow electrolyte to penetrate and accommodate mechanical strain [25]; (ii) well-developed porous carbon skeletons act as elastic buffers and conductive networks (especially during pyrolysis in inert/reductive gas flows) [26]; (iii) the large surface area with vast electrochemical active centres overcomes the limitation of primitive PBAs in reversible capacities [27]. Notably, in contrast with other MOFs (such as phenyl-based MOFs and zeolitic imidazolate frameworks), PBAs possess unique advantages including effective morphological modulation, environmental benignity, and promising economic efficiency when employed as templates [28, 29]. The regular morphologies (e.g. nanocubes and nanospheres) of PBAs can be perfectly retained after the thermal conversion process. PBAs are easy to prepare by room-temperature reaction in nontoxic aqueous solutions, thereby rendering PBA-templated synthesis an eco-friendly and economical approach compared with other templated synthetic methods. These features promote the broad development and employment of PBA derivatives in energy storage and conversion.

**Fig. 1:**
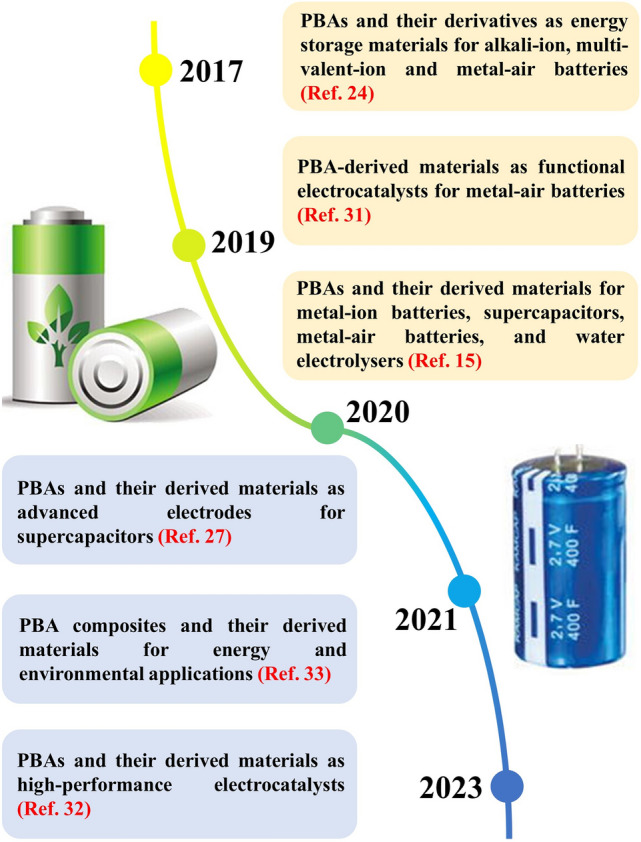
Schematic illustration for reviews about PBA-templated materials for electrochemical applications

**Fig. 2:**
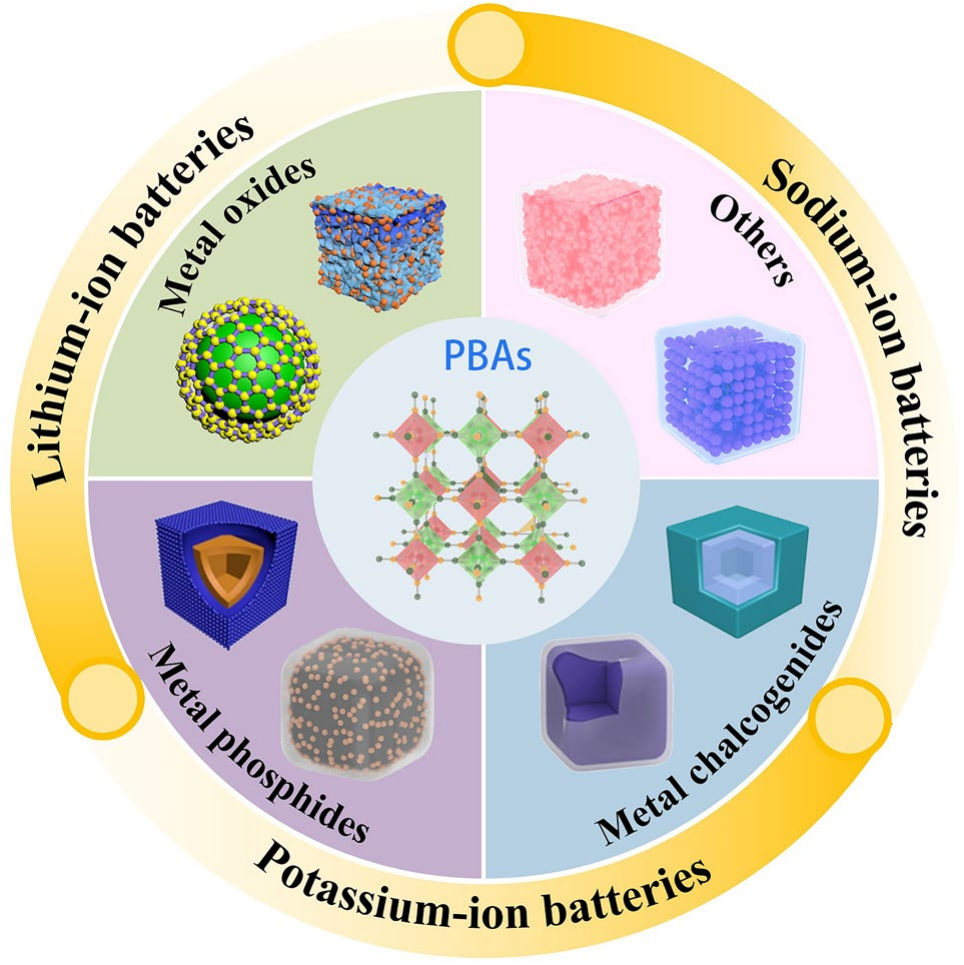
An overview of applications of PBA-templated nanomaterials in AIBs [34–41]. Copyright 2019, American Chemical Society; Copyright 2021, American Chemical Society; Copyright 2019, Wiley–VCH; Copyright 2020, American Chemical Society; Copyright 2024, Wiley–VCH; Copyright 2019, American Chemical Society; Copyright 2023, Wiley–VCH; Copyright 2023, Elsevier

Following the rapid advancement of PBA-templated nanocomposites, their applications in energy fields such as electrocatalysis [30–32], supercapacitors [27], and secondary batteries [24, 25, 33] have been widely reported. As presented in Fig. 1, the general discussion of PBA-templated materials in batteries and other electrochemical devices has been presented in some recent reviews, but more detailed excavation of their applications in AIBs is necessary. The systematic exploration of the synthesis, working mechanisms, and electrochemical behaviours of PBA-templated AIB electrodes can reinforce fundamental knowledge of alkali-ion storage with general features and instruct the directional design with a structure–composition–performance empirical link, thereby establishing a valuable platform for the innovation of the AIB technology with PBAs. Herein, this review begins with the synthetic protocol of PBA-templated nanocomposites from crystal growth to thermal conversion. The lithium/sodium/potassium storage mechanisms of these nanocomposites are comprehensively discussed in this work. Furthermore, the applications and electrochemical properties of various PBA-templated nanocomposites including metal oxides, metal chalcogenides, metal phosphides, and others in LIBs, SIBs, and PIBs are evaluated to outline their structure–activity correlation, as depicted in Fig. 2. Finally, perspectives of the future development direction of PBA-templated nanocomposites are proposed based on their merits and existing challenges, exhibiting more avenues for the next-generation battery technology.

## Preparation of PBA-Templated Nanocomposites

PBAs have been widely used in energy fields because of their abundant resource availability, easy preparation, intriguing redox activities, and framework structure ruggedness. From the industrial perspective, the facile preparation of PBAs is favourable for cost-effectiveness and eco-friendliness, rendering PBAs suitable self-sacrificial templates for the design of nanocomposites with desirable physicochemical properties. During the directional fabrication of these nanocomposites, crystal structure control, morphological engineering, surface modification, and superstructure development are crucial factors [42]. This section will provide an epitome for the bottom-up synthesis of PBA-templated nanocomposites from the PBA crystallisation to the structural evolution during thermolysis.

### Coprecipitation Method

The coprecipitation approach has been prevailingly used in PBA synthesis due to its simplicity and environmental benignity, during which the modulation of metal nodes, the selection of nucleation/crystallisation controlling agents, reaction time/temperature, solvent environment, etc. parameters are crucial to the resultant morphological and crystal characteristics. With a general chemical formula of A_*x*_M_*z*_[R(CN)_6_]_1–*y*_$\Upsilon$·*n*H_2_O (0 < *x* < 2; *y* < 1; A = alkali metals, M and R = transition metals, □$\Upsilon$ = R(CN)_6_ vacancy), PBAs show multifarious crystal phases, i.e. monoclinic, rhombohedral, cubic, tetragonal, and hexagonal structures due to the flexibly controllable composition [42, 43]. R sites in most PBAs are occupied by Fe, enabling the formation of a face-centred cubic structure with spacious interstitial spaces [19]. This enticing crystal structural property prompts the extensive attempts of fabricating cubic Fe-based PBAs, viz. FeFe-PB [40], NiFe-PBA [44], MnFe-PBA [45], and so forth by the facile coprecipitation route using transition metal salts and K_3_[Fe(CN)_6_]. It is worth noting that alkali metal sites show significant impacts on the resulting crystal structure, elucidating the phase transition upon the alkali-ion storage process in PBAs. For instance, Fe-based PBAs feature the typical cubic structure and may undergo crystal structural distortion from cubic to monoclinic with the increase in sodium content [46]. The morphological structure is another key parameter, which can be modulated by the variation in metal centres in transition metal cyanometalates. Aside from the prevailing cubic morphology, spherical/polyhedral morphological traits are available under proper conditions in Co-based PBAs, i.e. NiCo-PBA [47, 48], ZnCo-PBA [49], MnCo-PBA [35], and CoCo-PBA [50] synthesised by solution-based reactions between metal salts and K_3_[Co(CN)_6_]. The easy replacement of M and R sites endows PBAs with vast diversity in compositions and structures, thereby promoting the discovery and design of PBAs with multiple metal sites such as MnNiFe-PBA [51], high-entropy PBA with a single crystal structure (chemical composition: Na_1.70_Fe_0.2_Mn_0.2_Co_0.2_Ni_0.2_Cu_0.2_$\left[ {{\text{Fe}}\left( {{\text{CN}}} \right)_{{6}} } \right]_{0.98\Upsilon 0.02}$[Fe(CN)_6_]_0.98_□_0.02_·2.35H_2_O; abbreviated as SC-HEPBA) [22], NiFeCo-PBA [52], etc.)

External conditions are also instrumental to the structural features of PBAs, among which chelating agents interfere with the nucleation/crystallisation processes. The proper utilisation of chelating agents such as sodium citrate and polyvinyl pyrrolidone (PVP) warrants a slower nucleation process and controllable crystallisation, thereby resulting in regular morphologies [30, 46, 53]. By manipulating the reaction conditions, PBAs with similar chemical compositions may result in different morphological properties. Hydrothermal treatment is energetically favourable for defect engineering compared with the conventional room-temperature reaction, which was demonstrated in the preparation of Fe-PBAs [54]. Moreover, prolonged reaction time can facilitate the structural evolution from primitive solid architecture to 3D hierarchically hollow frame superstructure, which was validated by Lin et al. who prepared Fe–Co–Ni nanoframe superstructures (abbreviated as Fe–Co–Ni NFSs; chemical composition: Ni_3_[Fe_0.51_Co_0.46_(CN)_6_]_2_·6.56H_2_O) beginning with Fe–Co–Ni nanocubes synthesised by a typical coprecipitation process at room temperature [52]. These cases jointly substantiate the flexible and facile synthetic protocols of PBAs that ensure their structural designability and multiformity for energy applications.

### Post-Treatment Techniques

Based on the coprecipitation synthesis of pristine PBAs, PBA-based composites with more complex structures and dimensionality are accessible by adopting post-treatment techniques, i.e. electrospinning, template-assisted method, and surface coating. Electrospinning is a routine technique that helps fabricate MOF composites with 1D architectures and hence has been widely adopted in the fabrication of PBA composites [55, 56]. To develop a 1D network structure, CoFe-PBA cubic nanoparticles and polyacrylonitrile (PAN) were dispersed in *N,N′*-dimethylformamide (DMF) and then transferred to a syringe for electrospinning to obtain the CoFe-PBA@PAN composite [57]. Controlled crystal growth plays a crucial role in the formation of the 2D structure, which can be realised by employing hard/soft templates. Typically, graphene oxide (GO) is an ideal hard template extensively applied in the fabrication of 2D PBA/GO composites [58, 59]. The soft template-assisted method is applicable in some cases, during which feedstocks can precisely control the crystallisation orientation. Although most PBAs bear regular particle shapes, a 2D nanoplate structure was accessible by the fabrication of 2D Prussian green followed by the solution-based reaction [60]. Core–shell materials are propitious to stress buffering and hence extensively synthesised and investigated in energy storage, which remain designable by surface coating of PBAs, thereby motivating the fabrication of PBA composites with coating layers such as polydopamine (PDA) [37, 44] and resorcinol–formaldehyde (RF) [61]. The above techniques can effectively integrate PBAs with other components to acquire the desirable PBA composites.

Other than the integration of various components, in some cases, the removal or replacement of components was executed for structural and compositional optimisation by etching or ion-exchange reactions. By adjusting the solvent environment, the original morphological structures of PBAs can be partially devastated (especially in acidic or alkaline environments), thereby favouring the formation of void space. The existence of H^+^ facilitates the decomposition of [R(CN)_6_]^4–^, resulting in the formation of anion vacancies and voids [59]. In addition, a hollow cage morphology was designed by ammonia etching of the NiCo-PBAs [58], indicating the efficaciousness of alkaline conditions in morphological engineering. Therefore, the etching method using acidic or alkaline solvents can selectively remove the undesired components in PBAs to create more defects. When adopting this method to remove metal components, the chemical activity of metals is a crucial factor [62]. Active metal components (e.g. Zn, Co) can be easily removed with acid but acid etching is infeasible for the removal of inert metals (e.g. Cu). Furthermore, ion-exchange reactions are feasible for the replacement of components and have been employed for the substitution of anions and cations in PBAs [63]. The employment of post-treatment techniques distinctly extends the multiformity of PBAs, PBA composites, and their derivatives in dimensionality, morphology, and composition.

### Thermal Conversion

**Fig. 3:**
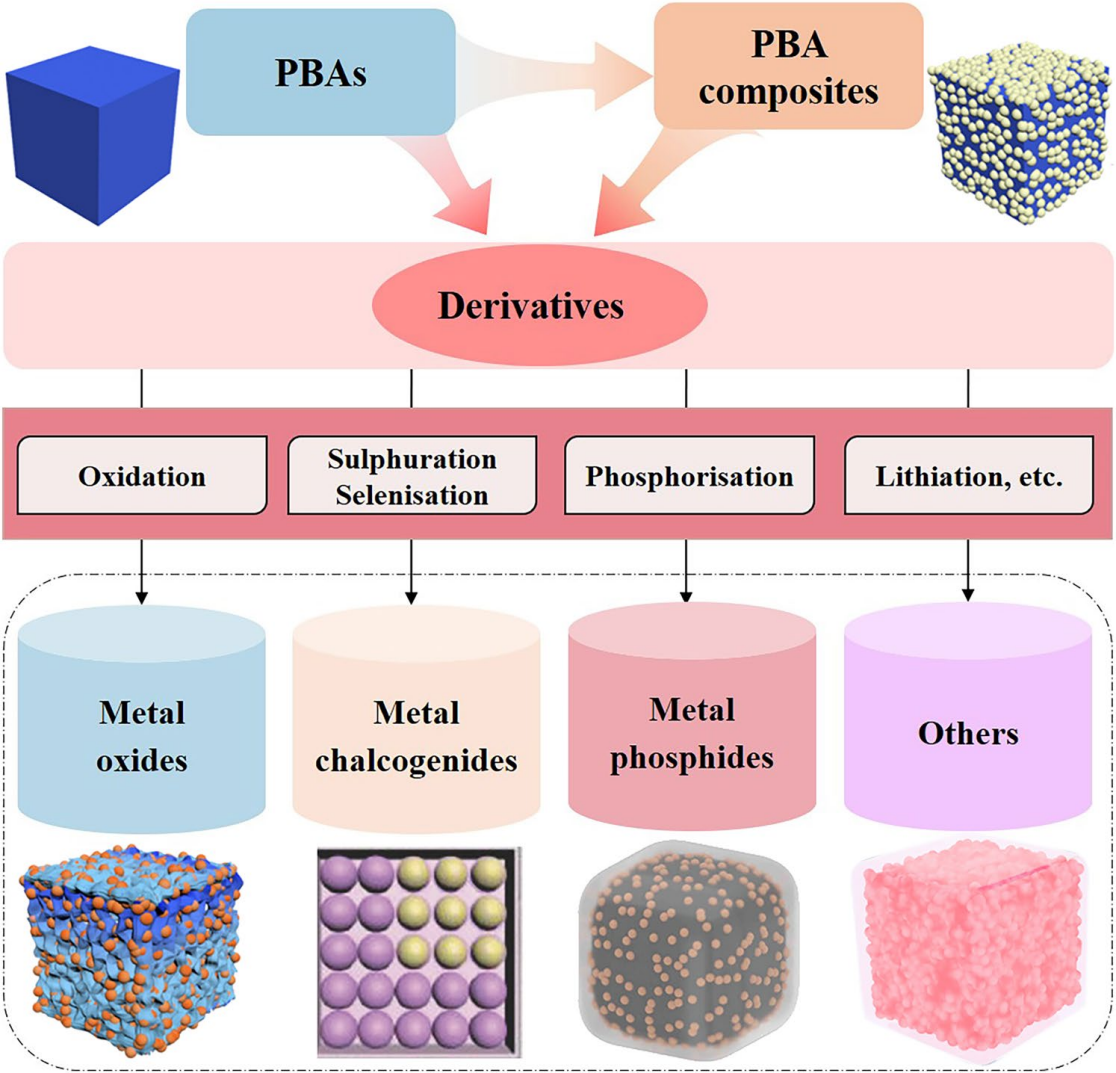
Bottom-up fabrication of PBA-templated nanocomposites [34, 38, 40, 74]. Copyright 2019, American Chemical Society; Copyright 2024, Wiley–VCH; Copyright 2023, Wiley–VCH; Copyright 2018, Wiley–VCH

The above synthetic protocols of PBAs and PBA-based composites establish a blueprint for the further design of PBA-templated nanocomposites including metal oxides, metal chalcogenides, metal phosphides, etc., as illustrated in Fig. 3. The in situ thermolysis of PBAs or their composites under proper conditions enables the successful synthesis of carbonaceous nanocomposites with desired compositional and morphological features, during which PBAs perform their dual functionalities as both precursors and templates. During the solid-state thermal conversion, the resulting nanostructures are highly correlated with the composition of self-sacrificial templates, reaction time/temperature, and gas atmosphere. Metal oxides (MOs) and their composites are accessible by direct thermolysis of PBAs and their composites under proper conditions, during which the Kirkendall effect related to the faster outward diffusion of metal ions than the inward diffusion of oxygen dominates the structural evolution and facilitates the formation of voids [40, 64, 65]. For instance, the calcination of the CoCo-PBA and its composites under the oxidation atmosphere enables the successful synthesis of Co_3_O_4_ or its carbon composite with affluent defect sites with the aid of the Kirkendall effect [64, 66]. The oxidation atmosphere also favours the formation of MOs with binary/multiple metal centres such as FeMnO_3_ derived from MnFe-PBA [67], FeMnO_3_/Mn_2_O_3_ nanocomposites derived from MnFe-PBA [68], and NiCo_2_O_4_ derived from NiCo-PBA [69] when using bimetallic PBAs as precursors. It is worth noting that porous carbon skeletons with N doping can be perfectly retained when calcined in reductive/inert atmospheres (N_2_, Ar, H_2_, etc.), which enables the fabrication of metal/carbon (M/C) composites [44, 62]. Motivated by the peculiar role of M/C composites as intermediates, other PBA-templated MO-based composites including metal oxide/carbon (MO/C) and metal/metal oxide/carbon (M/MO/C) are accessible by thermolysis in reductive/inert gas (e.g. Co_3_O_4_ nanoparticles embedded in N-doped carbon [66], FeMnO_3_ nanocages in N-doped carbon matrix [70], Co_3_O_4_/Co nano-heterostructures in N-doped carbon [71], and oxygen-deficient Co/MnO embedded in carbonaceous micropolyhedrons [35]). Many successful cases in the development of PBA-derived MOs and their composites establish a solid foundation for the nanostructuring of other metal compounds with PBAs, which endow the vast diversity of PBA-templated nanocomposites.

During the thermal decomposition process, one predominant advantage of the PBA-templated route is the easy introduction of heteroatoms internally or externally, which endows the accessibility of other PBA-derived materials including metal carbides, metal nitrides, metal sulphides, metal selenides, metal phosphides, metal phosphates, etc. Under a specific condition of 600 °C in N_2_ flows for 2 h, Co_3_ZnC species can appear during the thermolysis of CoZn-PBA [49, 72], where carbon originates from the cyano groups. PBA templates are also intrinsic nitrogen sources facilitating the formation of metal nitride phases that was validated by the in situ construction of (Ni/Co)_3_N multi-core nanoparticles dispersed in the N-doped carbon shell using the NiCo-PBA@PDA template [73]. Numerous examples reveal the great advantage of light element (e.g. N, C) doping when employing the PBA-templated approach. In contrast with conventional methods, this approach shows better executability because PBAs are effective light element donors and can result in successful doping by direct thermolysis. The utilisation of PBA templates also warrants the phase homogeneity of the resulting products and the even distribution of light elements (i.e. the homogeneous distribution of N in the carbon matrix) [40]. Apart from endogenous heteroatoms, exogenous heteroatoms (S, Se, P, etc.) can be imported by solution-based reactions or solid-state thermal conversion without severe devastation of primitive morphologies. To obtain PBA-templated metal sulphide-based nanocomposites, various S sources such as S powder [37, 57], H_2_S [48], Na_2_S [36], and thioacetamide (TAA) [61] were adopted during the direct/multi-step conversion. Likewise, metal selenide-based nanocomposites were conventionally acquired by the solid-state thermal treatment of PBAs/PBA composites and Se powder under inert atmospheres [74, 75]. As common AIB anode materials, metal phosphides and their composites were prepared by in situ phosphatisation of PBA precursors or their derived carbonaceous composites via thermal treatment with P sources (e.g. NaH_2_PO_2_·H_2_O and red phosphorous) [50, 76–78]. These cases jointly emphasise the executability and flexibility of heteroatom importing during the preparation of PBA derivatives, thereby prompting the design of AIB cathodes as Li/Na/K donors via the PBA-templated protocol. With the utilisation of Li sources (e.g. LiOH and Li_2_CO_3_), PBA-templated Li-based layered metal oxides such as LiCoO_2_ (LCO) [79] and LiNi_*x*_Co_*y*_Mn_*z*_O_2_ (NCM) [80] can be fabricated by thermal conversion at high temperatures. According to previous literature, the simultaneous use of NH_4_H_2_PO_4_ as the P source and Li_2_CO_3_ as the Li source contributed to the successful synthesis of lithium metal phosphate carbonaceous composites, during which N-doped carbon matrix inherited from PBA precursors constructed a conductive network [40, 41]. The high-temperature sintering strategy is also suitable in the preparation of K-based layered metal oxides as PIB cathodes, where K_2_CO_3_ or KOH was employed as K sources [81, 82]. The pronounced achievement in the PBA-templated synthetic route is an indispensable premise for their large-scale manufacturing and applications in AIBs.

### Analysis of Pros and Cons

**Fig. 4:**
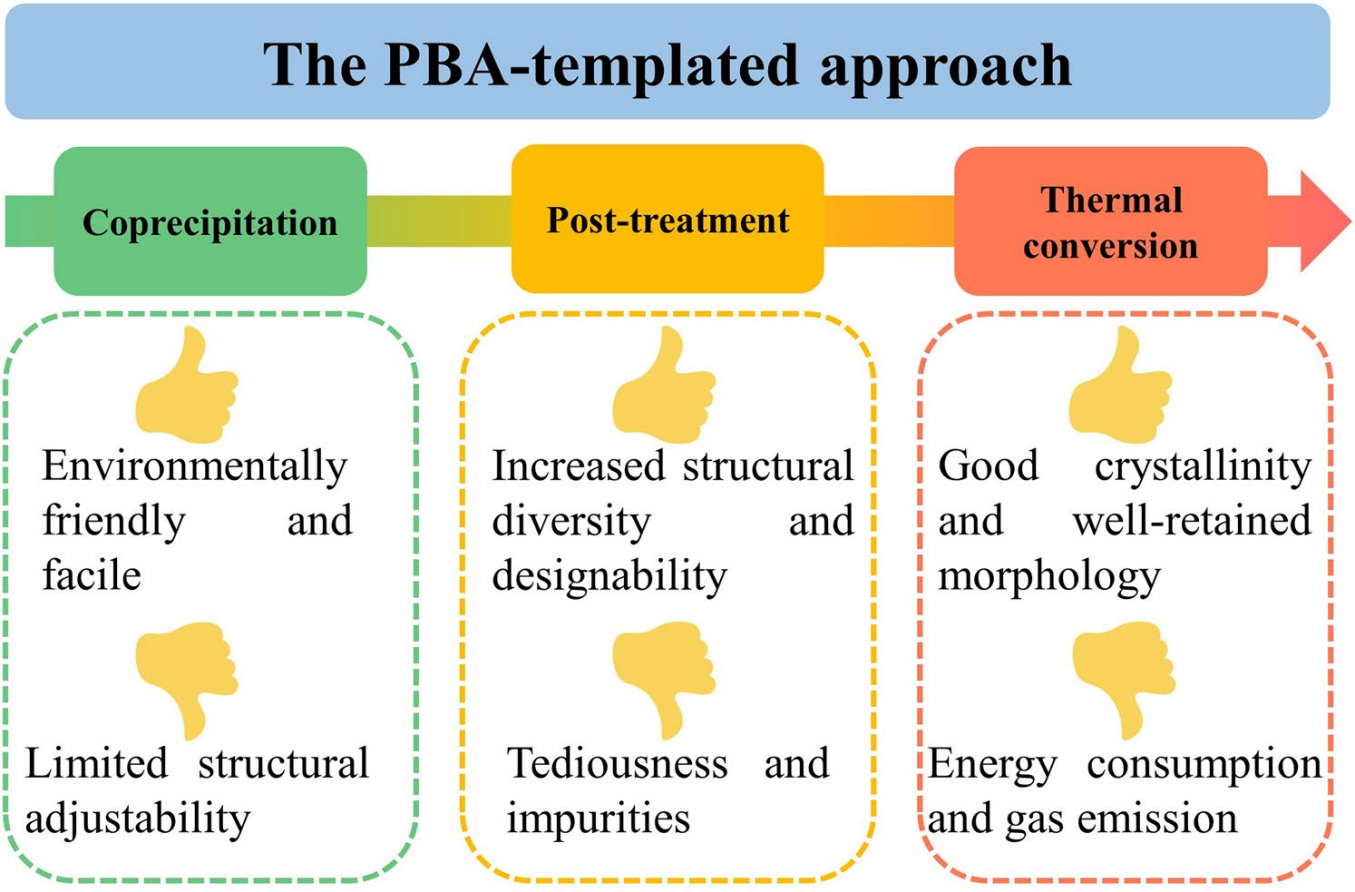
Advantages and disadvantages of synthetic techniques of PBA-templated nanocomposites

Both pros and cons are present in the PBA-templated approach, which are summarised in Fig. 4 to help further improvement in each synthetic procedure. The coprecipitation synthesis of PBAs is facile and green but usually results in limited structural adjustability. Although post-treatment techniques (electrospinning, templated assist methods, surface coating, etching, and ion-exchange reactions) can significantly increase the diversity of compositions and structures, they may make the synthetic route more tedious and import some undesirable impurities. The thermal conversion step can guarantee a good crystallinity degree and retain the parent morphology but is neither economical nor environmentally friendly due to high energy consumption and toxic gas emission. Therefore, the synthetic steps in the PBA-templated strategy still require further optimisation and simplification to satisfy the industrial need.

## Working Mechanisms of PBA-Templated Nanocomposites in AIBs

The PBA-templated functionalised nanocomposite electrodes of AIBs rely on intercalation, conversion, and alloying processes. These processes determine whether these nanocomposites have excellent lithium/sodium/potassium storage behaviours, whose comprehensive understanding will be insightful for the recognition of the pros and cons of different materials and in turn direct their targeted modification. Although the overall ion storage mechanisms of LIBs, SIBs, and PIBs are similar, the working voltage and specific electrochemical behaviours may vary due to the difference in the ion size, so it is necessary to distinguish the lithium/sodium/potassium storage mechanisms of PBA-templated nanocomposites for rational selection and matching of electrodes. With this aim, orchestrated endeavours have been devoted to mechanism analysis by in situ/ex situ characterisation when designing AIB electrodes. Hence, in this section, the working mechanisms of various PBA-templated electrodes in AIB systems are analysed by cases in detail.

### Lithium Storage

**Fig. 5:**
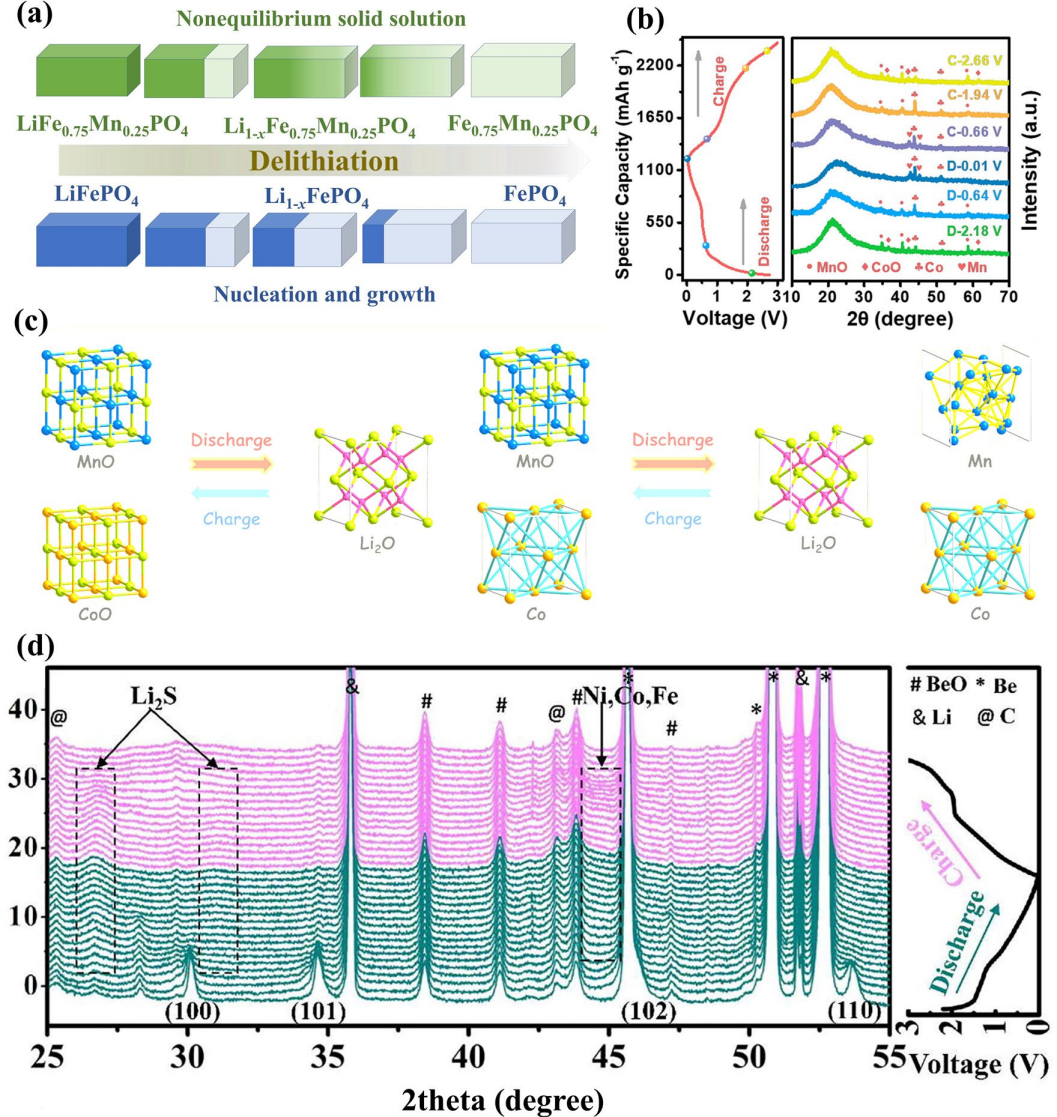
**a** Working mechanism of LFP cathodes with and without Mn doping. Reproduced with permission from Ref. [41], Copyright 2023, Elsevier. **b** Ex situ XRD patterns and **c** working mechanism of the MnO/Co anode material. Reproduced with permission from Ref. [35], Copyright 2021, American Chemical Society. **d** In situ XRD patterns of FeCo-NiS@NC. Reproduced with permission from Ref. [94], Copyright 2022, Elsevier

The commercialised LIB system in earlier days was composed of graphite anode and LiCoO_2_ cathode, where the lithium storage process relies on the repetitive lithium-ion intercalation/deintercalation and is defined as the “rocking chair” mechanism [83, 84]. As expected, layered LIB cathodes such as LiCoO_2_ [79] and NCM [80] derived from PBAs show typical intercalation mechanisms. Other than PBA-derived Li-based layered metal oxides, PBA-templated olivine cathode materials also deliver lithium storage capability relying on Li^+^ insertion/extraction accompanied by the transformation between Li-rich and Li-poor phases, which was systematically investigated recently [40, 41]. Notably, the lithium storage process of LiFePO_4_ (LFP) is correlated with the cation doping, as illustrated in Fig. 5a. The Mn-doped LFP bears a nonequilibrium solid solution formation process during the delithiation process, which is significantly different from the nucleation process of the primitive LFP and maintains the structural integrity upon cycling [41]. Apart from LIB cathodes, it is widely recognised that the graphite anode shows a layered structure allowing the intercalation process, which is expectedly feasible in PBA-derived porous carbon materials with similar lithium storage behaviours [85].

Despite the dominant position of the intercalation mechanism in practice, intensive attention has been dedicated to the exploitation of alloying-type and conversion-type LIB electrodes (especially anodes). PBA-derived nanocomposites containing alloying-type metal components such as ZnO/ZnFe_2_O_4_ hybrid nanostructures [86], ZnO/Co_3_ZnC/N-doped carbon composite [49], and ZnSe-Fe_3_Se_4_ heterostructures [75] present exceptional electrochemical performances in LIBs due to the reversible capacity provided by lithium metal alloys (e.g. LiZn_*x*_). The presence of conversion-type metal components results in the single/multi-step conversion mechanism, which occurs in other PBA derivatives such as Co_3_O_4_ [66, 87], Fe_*x*_Mn_2–*x*_O_3_ [68, 70], CoFeO_*x*_ [88, 89], FeS_2_ [61], Mn_0.6_Fe_0.4_S [90], CuFeS_2_ [91], CoP [92], and NiCoP [38]. These conversion reactions have been systematically explored and verified with sufficient evidence by in situ/ex situ characterisation. For instance, ex situ XRD patterns successfully unveil the reversible conversion between metal (Co and Mn) and metal oxides (CoO and MnO) when using the MnO/Co carbonaceous composite as a LIB anode (Fig. 5b, c) [35]. The MnO phase can be converted into Mn during the discharge process, after which MnO can be reversibly formed during the charging process. Meanwhile, the charging process enables the transformation from Co to CoO, while Co can be reversibly generated during the discharge process. The complementary lithium storage behaviours of MnO and Co result in the impressive reversible capacity of this composite, which is a typical example of the synergistic effect in lithium storage [93]. In another study, the conversion reaction of trimetallic metal sulphide composite, Fe/Co co-doped NiS with N-based carbon (FeCo-NiS@NC), was elaborately studied by the in situ XRD technique [94], where the generation of metal (Ni, Co, and Fe) and Li_2_S was identified (Fig. 5d). Upon lithiation, the FeCo-NiS phase can be converted into Ni, Co, Fe, and LiS, indicating the joint contribution of all metal centres and hence improving the overall performance. These findings support the extraordinary lithium storage capability of conversion-type materials derived from PBAs and convincingly substantiate their viability as advanced LIB anodes.

### Sodium Storage

As a pioneering rechargeable battery technology, SIBs have been regarded as suitable candidates to replace commercial LIBs, which show similar electrochemical behaviours based on intercalation/alloying/conversion reactions. Typically, pristine PBAs have been widely studied as SIB cathodes owing to their affluent redox-active centres, suitable operating voltages, and sufficient space for Na^+^ intercalation [23, 42]. As for anode materials, porous carbons with sufficient interlayer space are suitable intercalation-type anodes that allow the reversible insertion of more sodium ions in contrast with graphite [95, 96]. Despite the innumerable achievements in intercalation-type electrode exploration, the design of PBA-templated nanocomposites is still in its infancy due to the immaturity of synthetic protocols and a majority of these materials bear alloying/conversion mechanisms.

As mentioned above, the existence of alloying-type metals such as Zn will result in the formation of alloys, which is also applicable in sodium storage. When utilising the heterostructure ZnSe/FeSe nanospheres derived from Zn_3_[Fe(CN)_6_]_2_, both alloying and conversion reactions occur during the discharge process, as expressed below [53]:

1 $${\text{ZnSe }} + {\text{ 2Na}}^{ + } + {\text{ 2e}}^{-} \to {\text{Zn }} + {\text{ Na}}_{{2}} {\text{Se}}$$

2 $$x{\text{Zn }} + {\text{ Na}}^{ + } + {\text{ e}}^{-} \to {\text{ NaZn}}_{x}$$

3 $${\text{FeSe }} + y{\text{Na}}^{ + } + y{\text{e}}^{-} \to {\text{ Na}}_{y} {\text{FeSe}}$$

4 $${\text{Na}}_{y} {\text{FeSe }} + \, ({2}{-}y){\text{Na}}^{ + } + ({2}{-}y){\text{e}}^{-} \to {\text{ Na}}_{{2}} {\text{Se }} + {\text{ Fe}}$$

**Fig. 6:**
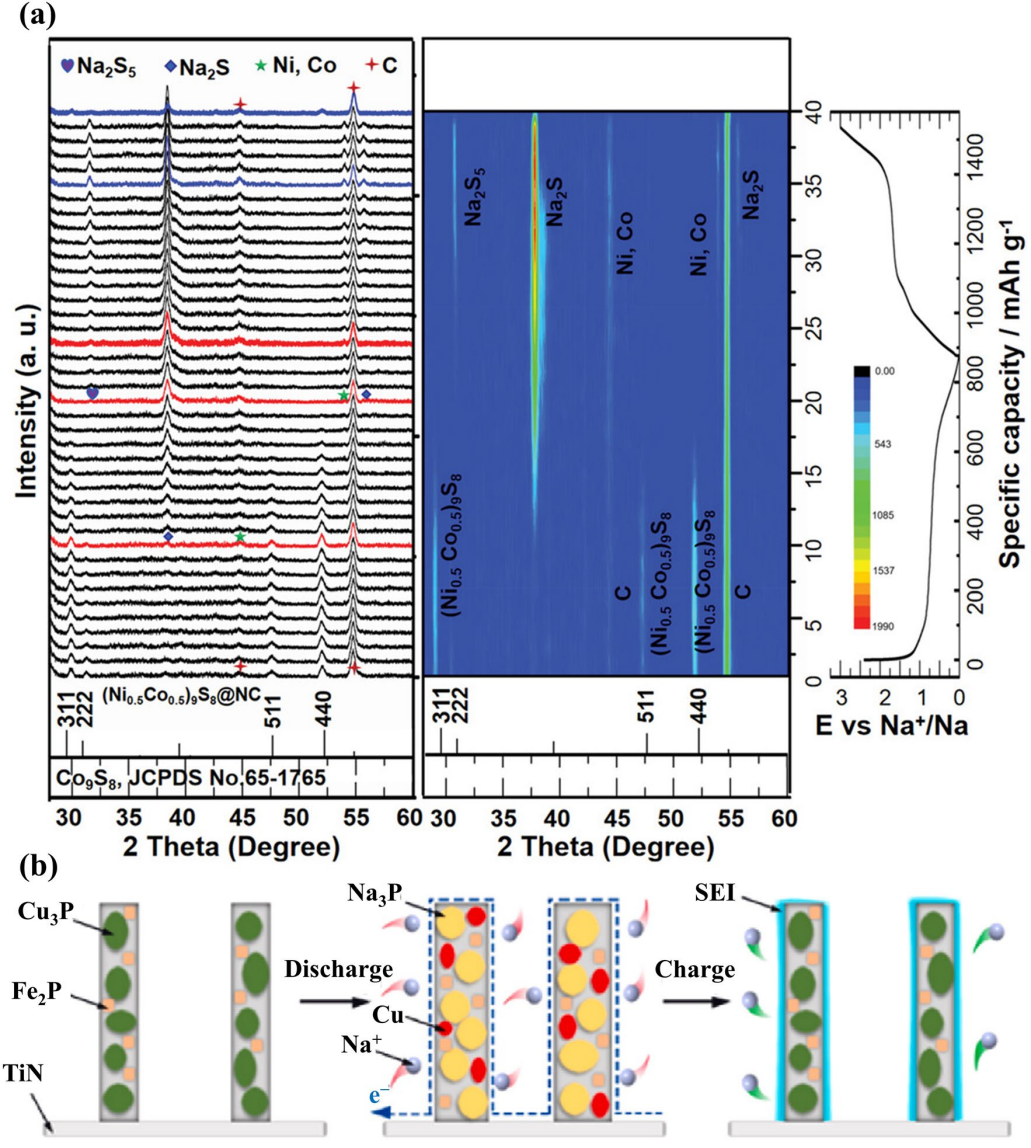
**a** In operando XRD results for (Ni_0.5_Co_0.5_)_9_S_8_@NC. Reproduced with permission from Ref. [36], Copyright 2019, Wiley–VCH. **b** The schematic illustration of the sodium storage process of the Cu-Fe–P/TiN anode. Reproduced with permission from Ref. [105], Copyright 2022, Elsevier

Without alloying-type components, most PBA-templated SIB anodes (i.e. CoFe_2_O_4_ [97, 98], MgFeO_2_ [99], Fe_7_S_8_ [100], MnS/FeS_2_ [37], (NiCo)S_4_ [101], MnS-CoS_2_ [102], CoS_2_/FeS_2_ [57], NiSe_2_ [103], CoSe_2_ [63], Ni_1.8_Co_1.2_Se_4_ [74], (Ni, Co)Se_2_ [58], FeP [104], and CoP/FeP [77]) contribute sodium storage capacity by single/multi-step conversion reactions. To reveal the conversion mechanism of NiCo bimetallic metal sulphide in N-doped carbon (abbreviated as (Ni_0.5_Co_0.5_)_9_S_8_@NC), in operando XRD analysis was executed, where characteristic peaks of Na_2_S, Na_2_S_5_, Co, and Ni were obtained (Fig. 6a) [36]. By the same technique, it demonstrated that the FeCu bimetallic phosphide can be converted to Cu, Fe, and Na_3_P upon discharge (Fig. 6b) [105], thereby emphasising the similarity of these materials in sodium storage. According to the above findings, it is noteworthy that the synergistic effect in sodium storage can also be observed in PBA-templated nanocomposites with multiple components.

### Potassium Storage

**Fig. 7:**
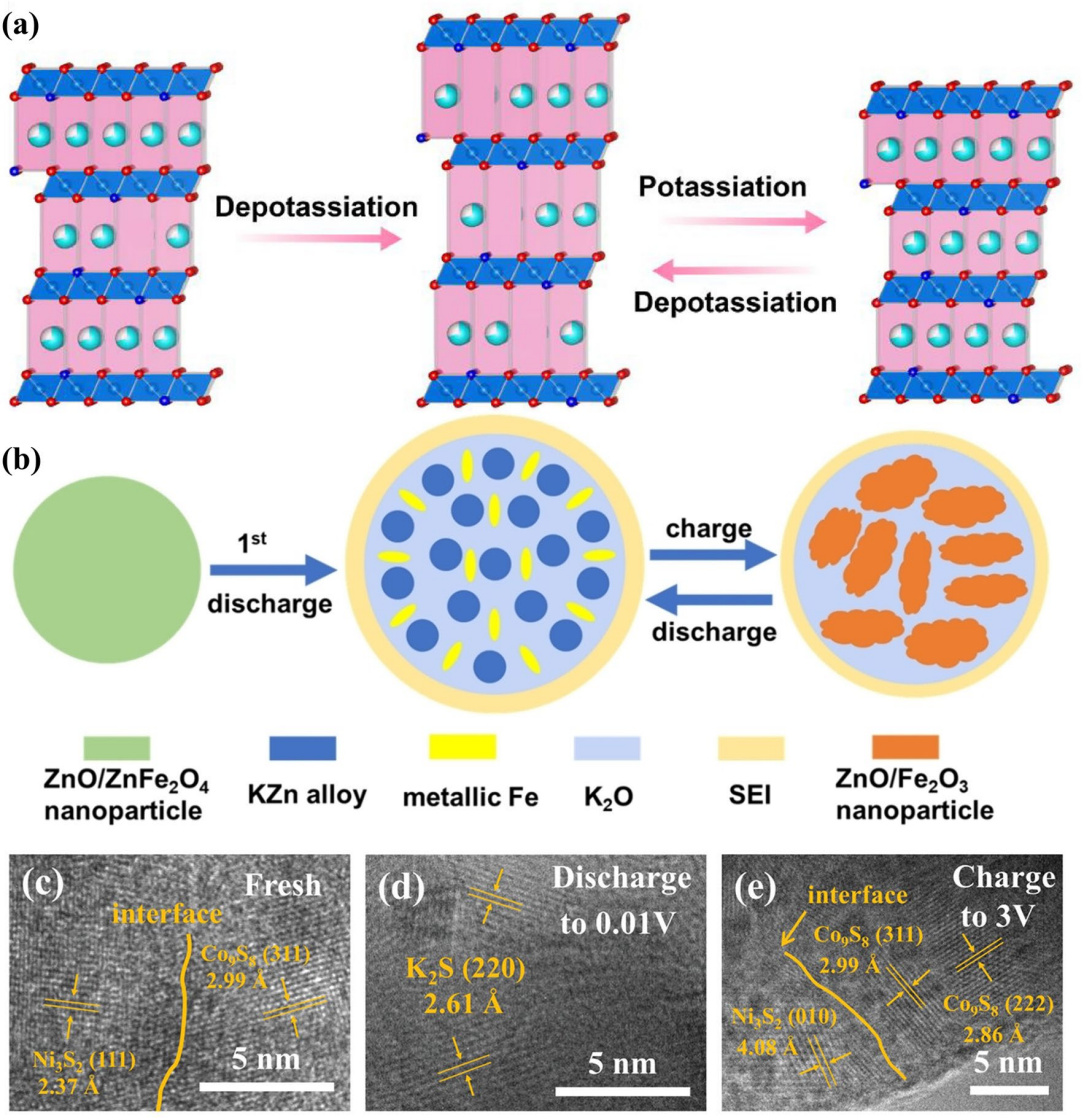
**a** Schematic diagram of potassium storage mechanisms for KMFON. Reproduced with permission from Ref. [82], Copyright 2023, Elsevier. **b** Alloying/conversion reactions of the ZnO/ZnFe_2_O_4_ PIB anode. Reproduced with permission from Ref. [107], Copyright 2023, Wiley–VCH. **c–e** Ex situ HRTEM images of Ni_3_S_2_–Co_9_S_8_ heterostructures. Reproduced with permission from Ref. [114], Copyright 2022, Wiley–VCH

The larger ion size of K^+^ compared with Na^+^ and Li^+^ requires more interstitial sites to afford the reversible insertion/extraction process, which has motivated the development of intercalation-type materials (layered metal oxides, PBAs, polyanion materials, porous carbons, etc.) [106]. Inspired by this, PBAs were utilised as templates to prepare other intercalation-type materials for better compositional and structural monitoring by integrating the intriguing merits of PBAs as morphological controllers and nitrogen sources. N-doped K-based layered metal oxides derived from PBAs can provide larger interlayer spacing for reversible K^+^ intercalation/deintercalation [81, 82]. As presented in Fig. 7a, the 2D layers provided by PBA-templated P3-typed K_0.5_Mn_0.67_Fe_0.33_O_1.95_N_0.05_ (KMFON) show amplified interlayer distance during depotassiation to allow K^+^ intercalation, resulting in remarkable reversibility with the negligible variation in the P3 phase. The reversible K^+^ insertion/extraction process and relatively high voltage plateau make K-based layered metal oxides suitable cathodes for PIBs. Nitrogen doping is conducive to elevating the electronic conductivity by forming a successive conductive network and hence is also feasible in porous carbons. Consequently, the PBA-derived Co nanoparticles in N-doped graphitised carbon (Co-NC) can deliver expedited kinetics during the intercalation process when investigated as the PIB anode [62].

Apart from intercalation-type PIB electrodes, PBAs were also utilised as self-sacrificial templates to design alloying/conversion-type materials for exalted potassium storage capacity. During the initial discharge process, ZnO/ZnFe_2_O_4_ nanoparticles can be transformed into KZn alloy, metallic Fe, and K_2_O, after which the reversible transformation occurs (Fig. 7b), as expounded below [107]:

5 $${\text{ZnFe}}_{{2}} {\text{O}}_{{4}} + {\text{ 9K}}^{ + } + {\text{ 9e}}^{-} \to {\text{ 4K}}_{{2}} {\text{O }} + {\text{ KZn }} + {\text{ 2Fe}}$$

6 $${\text{ZnO }} + {\text{ 3K}}^{ + } + {\text{ 3e}}^{-} \leftrightarrow {\text{ K}}_{{2}} {\text{O }} + {\text{ KZn}}$$

7 $${\text{Fe}}_{{2}} {\text{O}}_{{3}} + {\text{ 6K}}^{ + } + {\text{ 6e}}^{-} \leftrightarrow {\text{ 3K}}_{{2}} {\text{O }} + {\text{ 2Fe}}$$

PBA-derived conversion-type PIB anode materials (FeS_2_ [108], NiS/FeS [109], MnSe/FeSe_2_ [110], CoSe_2_/NiSe_2_ [111], CoSe_2_-FeSe_2_ [112], FeP [113], etc.) were also investigated due to their considerable reversible capacity induced by conversion reactions of electrochemical active components. Among them, nanocomposites with multiple active centres/components have gained particular attention due to the pursuit of the strengthened synergistic effect, where the synergistic effect refers to the complementary potassium storage behaviour of various components. For instance, the joint potassium storage behaviours of Ni_3_S_2_–Co_9_S_8_ heterostructures were unveiled by ex situ high-resolution transmission electron microscopy (HRTEM), as displayed in Fig. 7c–e [114]. When discharged to 0.01 V, the presence of the K_2_S phase indicated the reaction of K^+^ and primitive metal sulphide phases. It is noteworthy that Ni_3_S_2_ and Co_9_S_8_ can be reversibly generated when charged to 3 V suggesting that both of them were involved in conversion reactions and contributed to potassium storage capacity. With these convincing results, it can be assumed that the complementary effect of various components is beneficial to improving the overall potassium storage performance.

### Summary of Alkali-Ion Storage Mechanisms

Sufficient evidence has been proposed to validate the similarity of PBA-templated nanocomposites in lithium/sodium/potassium storage, during which various reactions corroborated by characterisations unearth root causes of lithium/sodium/potassium capacity and capacity attenuation of different materials. For intercalation-type materials, amplifying the interstitial space is an instrumental approach for adaptation to differences in ion radius, which is favourable for the reversible intercalation of more ions, thereby resulting in enhanced reversible capacities. Simultaneously, PBA templates enable atomic-level modulation and morphological engineering for ameliorated electronic characteristics and enlarged interlayer spacing for faster ion intercalation kinetics. Although intercalation-type materials are deemed to be suitable commercial electrodes for AIBs for their intrinsic safety, their lithium/sodium/potassium capacities are inadequate to satisfy future energy demands. Therefore, developing PBA template materials that work synergistically with other mechanisms is one of the effective ways to solve the above issue. Alloying-type materials exhibit substantial theoretical capacities due to the formation of metal alloys, but their huge volume change and low initial Coulombic efficiency are not satisfactory. Despite the considerable capacities induced by multi-electron reactions, conversion-type materials also show large irreversible capacities during the first loop and suffer from structural deterioration upon long cycling. To cope with these challenges, prelithiation/presodiation/prepotassiation techniques, nanonisation, and mechanical stress buffering are viable methodologies. Moreover, the development of heterostructures with hybrid lithium/sodium/potassium storage mechanisms remains an emerging trend, which helps to pursue the balanced merits of various components for strengthened synergistic effects.

Generally, intercalation, alloying, and conversion mechanisms of PBA-templated nanocomposites are similar to AIB electrode materials designed by conventional approaches [115–118], whose verification and identification can be useful fundamental knowledge for AIB electrode design using PBA-templated route. Based on these reactions, electrochemical reaction kinetics in AIBs can be quantified by pseudocapacitive studies and are vital to the Li^+^/Na^+^/K^+^ diffusivity. The cyclic voltammetry technique can be applied to estimate the pseudocapacitive contribution, which follows the formula [119]:

8 $$i_{{\text{p}}} = av^{b}$$

9 $$i\left( {\text{V}} \right) \, = k_{{1}} v + k_{{2}} v^{{{1}/{2}}}$$

In formula (8), *a* and *b* are constants, while *i*_p_ and *v* represent peak current and scan rate, respectively. In formula (9), *i*(V), *v*, *k*_1_*v*, and *k*_2_*v*^1/2^ stand for the current, scan rate, capacitance-dominated behaviour, and diffusion-controlled behaviour, respectively. To our best knowledge, the *b* value approaching 1 indicates the strong surface-induced capacitive process (SCP) or non-Faradaic process, while the one approaching 0.5 reveals the strong diffusion-controlled intercalation process (DIP) or Faradaic process. The SCP process indicates alkali-ion adsorption and is usually more palpable in conversion-type or alloying-type materials, while the DIP process implies alkali-ion intercalation and predominantly interferes with the alkali-ion storage of intercalation-type materials. In brief, the larger pseudocapacitive contribution ratio indicates faster ion/charge transfer and is conducive to the capacity utilisation rate at elevated current densities. Fortunately, the intrinsic open framework architecture of PBAs imparts their derived nanocomposites with sufficient void space after thermolysis. The crystal defects in PBA templates are common due to the absence of water molecules or organic ligands, which can also create vast defects in the resulting derivatives [24]. The existence of voids/defects facilitates SCP behaviours and hence is favourable for the SCP contribution of PBA-templated compounds during Li^+^/Na^+^/K^+^ storage [35, 120], thereby leading to their fabulous multiplier performance in AIBs. In contrast with the conventionally prepared AIB electrodes, the large surface area imparts PBA derivatives with abundant redox-active centres and ion adsorption sites [120], thereby resulting in strengthen ion storage reactions, ameliorated electrode/electrolyte contact, and more pronounced pseudocapacitive behaviours. The interstitial space is conducive to the adaptation of ion insertion and the alleviation of the mechanical stress caused by conversion/alloying processes. In addition, the effective nanostructuring induced by PBA templates can reduce the distance for alkali-ion migration and expedite the reaction kinetics. Therefore, the PBA-templated method can probably resolve the limitations and optimise the ion storage mechanisms of AIB electrodes prepared by conventional approaches without subverting routine electrochemical processes (intercalation/alloying/conversion reactions and pseudocapacitive/diffusion behaviours).

## Applications of PBA-Templated Nanocomposites in AIBs

The mechanism exploration of PBA-templated nanocomposites unambiguously elaborates their suitability as advanced AIB electrodes. Following the bottom-up synthetic method from controlled crystallisation of PBAs and their composites to solid-state thermal/solution-based conversion, multifarious PBA derivatives with vast multiformity in structures, compositions, and dimensionalities were meticulously designed. Despite the similarity in mechanisms, PBA-templated nanocomposites deliver different electrochemical performances in LIBs, SIBs, and PIBs, making it necessary to distinguish their applications in these AIB systems. The battery parameters like capacity, voltage range, and ion diffusivity are different in various AIBs, so the smart selection and optimisation of electrode materials can be vital to battery operation and performance [121]. Battery material design ought to be more connected to the full battery system in AIBs based on the systematic scientific analysis [122], which requires a comprehensive consideration of the applicability of different electrode materials. Hence, the particularities of PBA-templated nanocomposites in LIBs, SIBs, and PIBs need to be clearly identified. To highlight their structure–activity relationship in AIBs and in turn direct the monomer/condition selection, this section will categorise, compare, and discuss the lithium/sodium/potassium storage properties of various PBA-derived materials including metal oxides, metal chalcogenides, metal phosphides, and so forth.

### Lithium-Ion Batteries

The most commonly commercialised electrodes for LIBs play an important role in the energy market, but their lithium storage capacities are nearing their limits (theoretical capacity: ~ 372 mAh g^−1^ for graphite and ~ 148 mAh g^−1^ for LiCoO_2_) [123]. The quest for enhanced lithium storage properties offers opportunities for the ascension of PBA-templated nanocomposites with peculiar structural properties increasing lithium storage active sites. To understand the structure–activity correlation of these materials in LIBs and to guide their controlled synthesis, the lithium storage properties are summarised in Table 1.

**Table 1:** Lithium storage properties of PBA-templated nanocomposites

Electrodes	Templates	Initial DC/CC (mAh g^−1^)	Voltage (V vs. Li^+^/Li)	ICE (%)	RC/rate (mA g^−1^)	Cycle number	References
Co_3_O_4_	Co_3_[Co(CN)_6_]_2_	1499/1073	0.01–3.0	83.2	1229/50	20	[64]
Ag@Co_3_O_4_	CoCo-PBA	1291/1007	0.01–3.0	78	1015/200	100	[128]
NiCo_2_O_4_/NiO@C	CoCo-PBA@Ni-BTC	1415/1034	0.01–3.0	73.1	1433/1000	200	[132]
Co_3_O_4_ microcages@GA	CoCo-PBA@GA	2186/1514	0.01–3.0	69.3	1439/1000	200	[135]
Co_3_O_4_ microcubes@GA	CoCo-PBA@GA	1334/913	0.01–3.0	68.5	1235/1000	200	[136]
Co_3_O_4_@CNFs	CoCo-PBA@PAN	1113/864	0.01–3.0	95.8	1404/100	100	[66]
FeCo_2_O_4_	Co_3_[Fe(CN)_6_]_2_	1358/1074	0.01–3.0	79.1	1060/100	50	[124]
Co_x_Fe_3−x_O_4_	CoFe-PBA	1486/1435	0.01–3.0	96.6	1601/100	60	[126]
CoFeO_x_@C/C	Co_3_[Fe(CN)_6_]_2_	1043/676	0.01–3.0	58.7	483/5000	120	[88]
Fe-Co oxide@GA	FeCo-PBA@GA	1300/800	0.01–3.0	61	947/100	130	[134]
FCO@NC	FeCo-PBA	1230/798	0.01–3.0	~ 70	463/1000	500	[89]
NiO-Co_3_O_4_@rGO	NiCo-PBA	1640/991	0.01–3.0	60.5	805/100	100	[137]
Fe_2_O_3_@NiCo_2_O_4_	Ni_3_[Co(CN)_6_]_2_@Co_3_[Fe(CN)_6_]_2_	1311/903	0.01–3.0	68.8	1080/100	100	[127]
Mn_1.8_Fe_1.2_O_4_	Mn_3_[Fe(CN)_6_]_2_	2312/1337	0.01–3.0	57.8	827/200	60	[125]
FeMnO_3_	MnFe-PBA	2454/1418	0.01–3.0	57.8	710/100	200	[67]
FeMnO_3_/Mn_2_O_3_	Mn_3_[Fe(CN)_6_]_2_	1580/850	0.01–3.0	53.8	995/200	170	[68]
Fe-Fe_0.33_Mn_0.67_O/C	MnFe-PBA	1195/824	0.01–3.0	69	730/100	70	[131]
FeMnO_3_@NC	MnFe-PBA	1594/1167	0.01–3.0	~ 80	1011/500	100	[70]
ZnO/ZnFeO_4_	Zn_3_[Fe(CN)_6_]_2_	998/705	0.01–3.0	70.6	704/200	200	[86]
Fe_2_O_3_@CeO_2_	PB@CeO_2_	1351/986	0.01–3.0	72.8	886/100	100	[34]
ZnO/Co_3_O_4_	Zn_3_[Co(CN)_6_]_2_	2049/1164	0.01–3.0	56.8	957/100	100	[129]
Co_3_O_4_/N–C	Zn_3_[Co(CN)_6_]_2_	1200/840	0.01–3.0	~ 70	1255/100	100	[87]
NiFe_2_O_4_-FeMnO_3_	Ni_*x*_Mn_*y*_[Fe(CN)_6_]_2_	1698/957	0.01–3.0	56.4	536/500	500	[130]
O_v_-MnO/Co NCPs	MnCo-PBA	2124/1638	0.01–3.0	77.1	1349/1000	1000	[35]
FeVO_4_@CNTs	FeV-PBA	475/250	0.01–3.0	52.6	400/250	150	[133]
FeS_2_@C	FeFe-PB	963/650	0.01–3.0	67.5	560/100	100	[61]
Fe_7_S_8_@NC@MoS_2_	FeFe-PB	−	0.01–3.0	−	1000/100	100	[100]
NiS_2_@CoS_2_@C@C	NiCo-PBA	1380/988	0.01–3.0	71.6	680/100	100	[138]
C-coated KCuFeS_2_	CuFe-PBA	−	0.01–3.0	~ 85	380/250	500	[91]
Mn_0.6_Fe_0.4_S	MnFe-PBA	809/612	0.01–3.0	76	520/1000	1000	[90]
FeCo-NiS@NC	FeCoNi-PBA	1179/1010	0.01–3.0	85	514/2000	600	[94]
CoP@GA	CoCo-PBA	1213/1075	0.01–3.0	88.6	805/200	200	[92]
CoP/Co_2_P@N–C	CoCo-PBA	1440/951	0.01–3.0	66	978/100	100	[50]
NiCoP@N–C	NiCo-PBA	1258/846	0.01–3.0	67.3	859/100	120	[38]
Co_3_ZnC/C	Zn_3_[Co(CN)_6_]_2_	908/607	0.01–3.0	66.9	608/100	300	[72]
ZnO/Co_3_ZnC/NC	Zn_3_[Co(CN)_6_]_2_	1164/744	0.01–3.0	64	1162/200	300	[49]
(Ni/Co)_3_N MC@HC	Ni_3_[Co(CN)_6_]_2_	−	0.01–3.0	−	440/200	130	[73]
NCCH	NiCo-PBA	−	0.01–3.0	−	437/200	300	[85]
LiCoO_2_	Co_3_[Co(CN)_6_]_2_	−	3.12–4.3	−	102/274	100	[79]
NCM	(Na_0.25_K_0.15_)-Ni_2.6−x_Mn_x_[Co(CN)_6_]_2_	222/190	2.7–4.5	85.6	152/140	100	[80]
LFP/CN	FeFe-PB	−	2.3–4.8	−	153/85	500	[40]
LiMn_0.25_Fe_0.75_PO_4_/C	MnFe-PBA	−	2.3–4.8	−	165/85	200	[41]

DC/CC: discharge/charge capacity, ICE: initial Coulombic efficiency, RC: reversible capacity

#### Metal Oxides

Metal oxides, due to their enticing theoretical capacities, have been extensively reported as potential alternatives for conventional graphite. Nevertheless, the structural frangibility and severe volume variation cause drastic capacity attenuation, thereby prompting the adoption of PBA templates to obtain desired nanostructures and realise structural/compositional optimisation. It is widely acknowledged that hollow architectures with affluent voids can help stress buffering and facilitate Li^+^/electron transportation, motivated by which the direct thermolysis of PBAs in air was executed in earlier days, resulting in numerous metal oxides with nano/microstructures such as hollow FeCo_2_O_4_ nanospheres [124], porous Mn_1.8_Fe_1.2_O_4_ nanocubes [125], Co_3_O_4_ porous polyhedrons [64], and FeMnO_3_ microcubes [67]. These PBA-derived metal oxides with single component presented incredible initial capacities (> 1000 mAh g^−1^) but couldn’t satisfy practical battery systems due to their bad cycling lifespan (less than 100 cycles) and dissatisfying initial Coulombic efficiency. For structural optimisation, the annealing temperature is crucial, which was systematically investigated when synthesising CoFe-PBA-templated Co_*x*_Fe_3−*x*_O_4_ nanocubes [126]. The decomposition of cyano groups in the precursor promotes the interdiffusion process accompanied by the formation of the hierarchically porous structure with excellent phase homogeneity, making 700 °C the optimal temperature with superior reversible capacities (1601 and 950 mAh g^−1^ at 100 and 5000 mA g^−1^, respectively) and elucidating the significance of processing conditions.

To enhance structural integrity and alleviate mechanical stress, researchers have designed core–shell structures with multiple components. The calcination of Ni_3_[Co(CN)_6_]_2_@Co_3_[Fe(CN)_6_]_2_ was employed to develop Fe_2_O_3_@NiCo_2_O_4_ porous nanocages, during which the Kirkendall effect played a part in the formation of the hollow cubic architecture [127]. Due to the synergistic effect and the well-developed porosity, this composite anode exhibited a splendid initial capacity of 1311.4 mAh g^−1^ at 100 mA g^−1^ with a retention rate of 82.3% after 100 cycles. In some cases, epitaxial shells of core–shell structures can efficaciously maintain the structural integrity of bulk materials and boost the overall electrical conductivity. It is reported that the uniform deposition of CeO_2_ on the Prussian blue (PB) precursor and the thermolysis at 400 °C resulted in the successful fabrication of Fe_2_O_3_@CeO_2_-400 LIB anode with superior rate capability (643 mAh g^−1^ at 1000 mA g^−1^) and cyclability (886 mAh g^−1^ at 100 mA g^−1^ for 100 cycles) [34], which can be accredited to the enhanced thermal stability and boosted Li^+^ diffusion. To incorporate the advantages of interior cavities and surface conductive layers, Ag nanoparticles were uniformly deposited on the surface of PBA-derived Co_3_O_4_ hollow nanoboxes, resulting in an outstanding reversible capacity of 1015 mAh g^−1^ after 100 rounds at 200 mA g^−1^ and considerable capacity retention of 361 mAh g^−1^ at 5000 mA g^−1^ [128]. Although Ag coating pronouncedly exalted the electrochemical performance of bare Co_3_O_4_ electrodes, this strategy hardly meets the commercial requirement due to economic considerations.

Coupled with innumerable experimental results, it can be assumed that the joint contribution of various components can maximise the lithium storage capability, which has inspired the synthesis of heterostructural nanocomposites, viz. ZnO/Co_3_O_4_ nanocomposite clusters derived from Zn_3_[Co(CN)_6_]_2_ nanospheres [129], ZnO/ZnFe_2_O_4_ hybrid nanostructures derived from ZnFe-PBA [86], FeMnO_3_/Mn_2_O_3_ hybrids derived from Mn_3_[Fe(CN)_6_]_2_·*n*H_2_O [68], and NiFe_2_O_4_-FeMnO_3_ derived from Ni_*x*_Mn_*y*_[Fe(CN)_6_]_2_ [130]. In the above Zn-based nanocomposites, ZnO is not only an electrochemically active component with an alloying mechanism contributing lithium storage capacity but also a buffer domain in the heterostructure inhibiting self-aggregation, resulting in more stable cycling performances (> 900 mAh g^−1^ at 100 mA g^−1^; > 700 mAh g^−1^ at 200 mA g^−1^). The impact of chelating agents on resulting morphologies was validated during the preparation of FeMnO_3_/Mn_2_O_3_ hybrids, whereas citric acid can facilitate the formation of Mn_2_O_3_ nanotubes with amplified active sites and hence enhance the reversible capacity (~ 1000 mAh g^−1^). Compositional optimisation is another crucial consideration during heterostructure design that was examined by adjusting the Ni/Mn ratio in NiFe_2_O_4_-FeMnO_3_ (536.1 mAh g^−1^ at 500 mA g^−1^ over 500 cycles when Ni: Mn = 2: 8) [130]. Therefore, both morphological and compositional properties are instrumental factors for the lithium storage behaviours of PBA-derived nanocomposites.

**Fig. 8:**
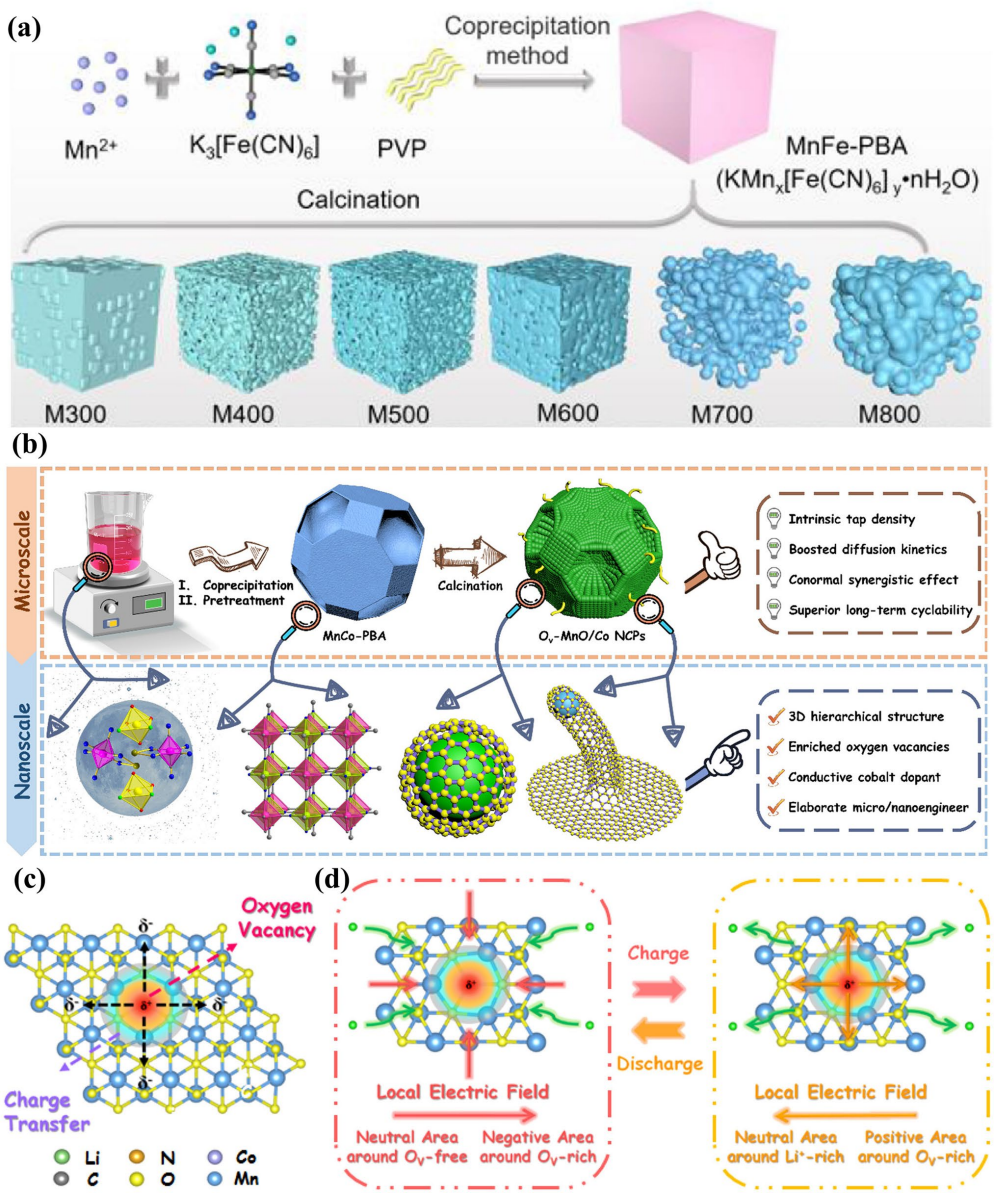
**a** PBA-templated synthesis of manganese-iron oxide-based carbonaceous hybrids from 300 to 800 °C. Reproduced with permission from Ref. [131], Copyright 2023, Elsevier. **b** Synthesis route, **c** charge transfer phenomenon around oxygen defect sites, and **d** boosted lithium diffusion related to the locally built-in electric fields for O_v_-MnO/Co NCPs. Reproduced with permission from Ref. [35], Copyright 2021, American Chemical Society

According to the above findings, although thermal decomposition in an oxidation atmosphere usually results in excellent crystallisation orientation and enticing hollow structures with perfect retention of parent morphologies, the fatal demerit of subpar electronic conductivity of metal oxides needs to be reconciled, thereby inspiring the manufacturing of metal oxide carbonaceous composites by solid-state conversion of PBAs under reductive/inert atmospheres. One facile method to generate carbon matrices is the direct thermolysis of PBAs in anoxic circumstances, where the PBA templates are ideal carbon sources. By a two-step calcination process, a ZnCo-PBA-derived composite with Co_3_O_4_ nanocrystals in N-doped carbon (Co_3_O_4_/N–C) was fabricated, during which the volatilisation of Zn and oxidation of Co generated vast defects and endow Co_3_O_4_/N–C with a fabulous lithium storage capacity of 1255 mAh g^−1^ significantly outperforming the carbon-free counterpart [87]. As mentioned earlier, the resulting microstructure is correlated with the calcination temperature, which was systematically explored by using MnFe-PBA as the self-sacrificial template to synthesise manganese-iron oxide-based carbonaceous hybrids [131]. As shown in Fig. 8a, the primitive cubic structure can be well inherited below 700 °C, while irregular agglomerates appear over 700 °C. The composition of resultant products also varies from 300 to 800 °C (MnO_2_–Mn_2_O_3_/C, MnO_2_–Fe_0.33_Mn_0.67_O/C, Mn-Fe_0.33_Mn_0.67_O/C, Fe–Fe_0.33_Mn_0.67_O/C, Fe_3_Mn_3_O_8_–Fe_0.33_Mn_0.67_O/C, and MnO–Fe_3_C–Fe/C) emphasising the significance of the calcination temperature in structural/compositional manipulation. Among these products, Fe-Fe_0.33_Mn_0.67_O/C showed superiority in rate capability (589.2 mAh g^−1^ at 1600 mA g^−1^) and cycling stability (626.8 mAh g^−1^ at 1000 mA g^−1^ for 1000 cycles) owing to the enticing porous structure with abundant void space, Fe nanoparticles kinetically accelerating the electrochemical reactions, and N-doped carbon layer acting as an elastic buffer for volume fluctuation. The meticulous control of thermal treatment can result in the presence of metal nanoparticles in the resultant nanocomposites with additional lithium storage capability. These nanocomposites derived from PBAs with the optimal composition possess increased active centres and ameliorated electrode/electrolyte contact but are inclined to suffer from particle agglomeration and display inferior tap density, which are insufficient to satisfy practical use and stimulate the multiscale modification of hierarchical architectures to integrate the merits of nanoengineering and microengineering. By calcination of MnCo-PBA at 700 °C, a hierarchical composite with oxygen vacancy (O_v_)-rich MnO/Co nanoparticles embedded in N-doped carbon nanotube-assembled carbonaceous micropolyhedrons (O_v_-MnO/Co NCPs) was obtained, which delivered enhanced tap density compared with conventional nanocomposites due to the unique 3D hierarchical structure (Fig. 8b) [35]. Consequently, this hierarchical material displayed splendid long-term cyclability at different operating conditions (1349.1 mAh g^−1^ at 1000 mA g^−1^ for 1000 cycles; 1106.2 mAh g^−1^ at 2000 mA g^−1^ for 800 cycles at 60 °C) due to the rationally designed structure, a strong synergistic effect between various components, and the affluent O_v_ amending atomic/electronic structure. Notably, the imbalanced charge distribution and charge transfer behaviour usually appear around O_v_ sites (Fig. 8c), thereby resulting in the expedited Li^+^ mobility during charge/discharge processes induced by the local electric fields (Fig. 8d) and revealing the feasibility of O_v_ engineering.

To our best knowledge, carbon modification is a productive methodology to enhance electrical conductivity for a higher capacity utilisation rate (particularly at elevated current densities). This can be achieved not only by retaining the porous carbon skeletons of PBAs but also by employing an external carbon source. For instance, the surface coating strategy was implemented using carbon sources such as polypyrrole (PPy) [70] and PDA [89]; then, by the calcination procedure the PBA-derived metal oxides were successfully coated by carbon to prepare the FeMnO_3_@NC composite and N-doped carbon-coated FeO/CoO hollow nanocages (FCO@NC), respectively. As expected, this synthetic route fully utilises both the abundant void space induced by the decomposition of PBAs in air and the N-doped carbon phase as the protective layer, simultaneously enhancing the structural robustness and boosting the electron transfer, thereby resulting in the superior lithium storage capability of these carbonaceous products compared with their counterparts without carbon coating. Similar to PBAs, other MOFs are also promising carbon sources, inspired by which a dual template strategy was proposed to fabricate N-doped carbon-coated NiCo_2_O_4_/NiO nanorods (denoted as NiCo_2_O_4_/NiO@C) [132]. The hybrid MOF, CoCo-PBA@Ni-BTC, was hydrothermally synthesised, whose hierarchical urchin-like architecture warranted the large surface area, shortened ion/electron transfer paths, and alleviated volume expansion of the resulting product, thereby contributing to the stable reversible capacity of 1432.8 mAh g^−1^ with a Coulombic efficiency of 98%. Moreover, the thermal treatment process under inert/reductive circumstances energetically facilitates oxygen defect formation to boost Li^+^ diffusion kinetics. Motivated by this, CoFe-PBA was deposited on a 3D carbon network and then underwent an oxidation-thermolysis process to yield a double carbon-reinforced defective metal oxide composite (abbreviated as CoFeO_x_@C/C) [88]. This double carbon modification strategy endows CoFeO_x_@C/C with intriguing capacity retention of over 85% at 200 mA g^−1^ for 120 cycles and negligible capacity fade during the rate performance measurement (906.1 and 839.8 mAh g^−1^ at 100 and 1000 mA g^−1^, respectively). The construction of successive conductive networks can favour surface defect formation towards a more predominant capacitive-controlled process, which can be realised by embedding metal oxide nanoparticles on carbon nanofibers (CNFs) or carbon nanotubes (CNTs). Motivated by this, a two-step thermal treatment process was executed to obtain Co_3_O_4_@CNFs after the formation of the Co-PBA@PAN composite by electrospinning [66]. In another work, FeV-PBA-derived Fe_2_VO_4_ nanoparticles were integrated with CNTs by sonication in ethanol [133]. These 1D composites also performed well when investigated as LIB anodes due to the reduced Li^+^ diffusion distance and the unimpeded electron transfer pathways.

Despite the predominantly enhanced lithium storage properties induced by the carbon matrix/network, the fluffy structure with more electrochemically inert components is prone to taper the volumetric energy density. Therefore, the exploitation of binder-free electrodes remains an emerging trend for the future LIB industry, during which conductive hard substrates with small volume and light weight have been widely adopted to enhance both structural rigidity and conductivity. Considering the excellent structural robustness of graphene aerogel (GA) as a 3D conductive substrate, GA encapsulated metal oxide composites derived from PBAs such as Fe-Co oxide@GA [134], Co_3_O_4_ microcages@GA [135], and Co_3_O_4_ microcubes@GA [136] were developed by hydrothermal growth of PBAs on GO, freeze drying, and calcination. The proper ratio of GA productively mitigated particle agglomeration and structural deterioration during cycling to obtain outstanding high-rate performance (> 1000 mAh g^−1^ at 1000 mA g^−1^ for 200 cycles). Recently, reduced graphene oxide (rGO) encapsulation of NiO/Co_3_O_4_ derived from NiCo-PBA was also proposed, by which the volume fluctuation can be well resolved by the integration of hollow structure and rigid conductive network, thereby resulting in stable lithium storage capacity [137]. Notable achievements in PBA-templated metal oxide-based nanocomposites signify the viability of PBAs in the controlled synthesis of these materials from macroscopic to microscopic levels.

#### Metal Chalcogenides

Metal chalcogenides (metal sulphides and metal selenides) show similar lithium storage behaviours and have attracted board attention due to their boosted electronic/ion conductivity and enhanced thermal stability compared with metal oxides. The significant volumetric change and structural brittleness of metal sulphides severely hamper their practical application, which requires structural engineering to tolerate the volume fluctuation upon cycling and hence provides opportunities for PBA-templated synthetic routes. As mentioned earlier, PBA-derived core–shell structures can afford volume expansion and mechanical stress towards higher structural integrity. To develop unique core–shell FeS_2_@carbon (FeS_2_@C) spheres, RF coating was executed by the solution-based reaction to obtain FeFe-PB@RF, after which thermal treatment was implemented to acquire the Fe_2_O_3_@C intermediate and then the solvothermal sulphurisation with TAA as the sulphur source successfully converted the intermediate into FeS_2_@C [61]. The primitive core–shell structure was well retained after this multi-step synthesis, which resulted in stable capacities in LIBs (560 mAh g^−1^ at 100 mA g^−1^ for 100 cycles; 269 mAh g^−1^ at 1000 mA g^−1^ for 500 cycles).

**Fig. 9:**
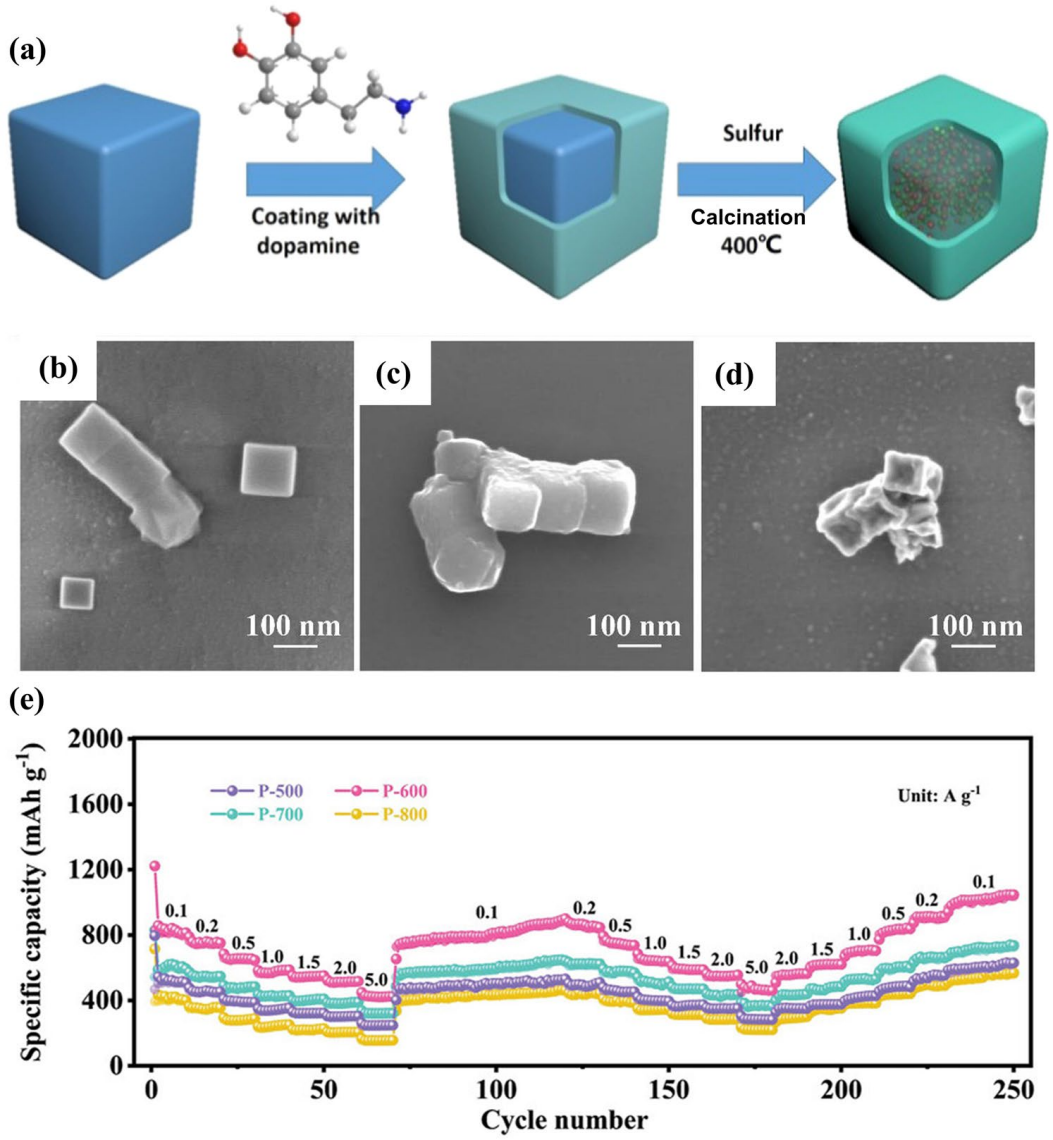
**a** Synthetic route of NiS_2_@CoS_2_@C@C. Reproduced with permission from Ref. [138], Copyright 2018, Elsevier. **b–d** Scanning electron microscopy (SEM) images of NiCoFe-PBA, NiCoFe-PBA@PDA, and FeCo-NiS@NC. Reproduced with permission from Ref. [94], Copyright 2022, Elsevier. **e** Rate capability of NiCoP@N–C materials prepared at different temperatures. Reproduced with permission from Ref. [38], Copyright 2023, Wiley–VCH

The above result experimentally validated the efficaciousness of core–shell structure engineering in the modification of single-metal sulphides, which is also applicable in other bi/multi-metallic sulphides derived from PBAs. For instance, after the solid-state conversion of NiCo-PBA@PDA and sulphur at 400 °C, double carbon-wrapped NiS_2_@CoS_2_ hetero-nanocrystals (NiS_2_@CoS_2_@C@C) were obtained [138], as illuminated in Fig. 9a. The hierarchical porosity and double carbon frameworks allowed the reversible lithium storage process and resulted in a negligible capacity attenuation (680 mAh g^−1^ at 100 mA g^−1^ for 100 cycles). To prevent structural disintegration and particle agglomeration, the coating strategy was also applied in the modification of other bi/multi-metallic metal sulphides. According to the previous report, the in situ polymerisation of PDA on the surface of CuFe-PBA enabled particle size control and prevention of particle agglomeration during the thermal sulphuration process, which kinetically favoured the lithium storage properties of the carbon-coated KCuFeS_2_ electrode (800 mAh g^−1^ at 10 mA g^−1^) [91]. The intriguing performance of these sulphide-based nanocomposites is attributed to not only the conductive carbon layer but also the incorporation of active components. Typically, the solid solution mechanism is propitious to a better electronic structure and can facilitate the charge transfer process, which was substantiated in the cubic Mn_0.6_Fe_0.4_S material with the FeS_2_/MnS solid solution during the thermal sulphurisation process [90]. Interfacial properties in heterostructures also affect the Li^+^ diffusion kinetics, which can maximise the functionality of individual components. The delicately designed hybrid with MoS_2_ grown on N-doped carbon-coated Fe_7_S_8_ (Fe_7_S_8_@NC@MoS_2_) delivered expedited lithium storage kinetics owing to the controllable voids between the inner core and outer shell and the sufficient active area in the interface induced by MoS_2_ coating [100]. The joint functionalities of various active centres are crucial for the resulting capacity, motivated by which the metal sulphide composite with multiple metal centres was designed. As portrayed in Fig. 9b–d, the primitive cubic architecture was hardly devastated during the synthesis of Fe, Co–co-doped NiS in N-doped carbon (FeCo-NiS@NC) from the PDA surface coating of NiCoFe-PBA to the thermal decomposition of NiCoFe-PBA@PDA [94]. Remarkably, the rationally designed structure and modulation of metal element ratio imparted FeCo-NiS@NC with suitable working voltage and stable capacity at a harsh condition (514 mAh g^−1^ at 2000 mA g^−1^ for 600 cycles), thereby emphasising the significance of morphological and compositional design for PBA-templated metal sulphides.

#### Metal Phosphides

Metal phosphides bear dazzling theoretical capacities (~ 900 mAh g^−1^) but are subjected to structural vulnerability and poor electrical conductivity. Carbon modification methods can effectively mitigate these drawbacks, suggesting the feasibility of PBA-derived synthetic routes due to the retainable porous carbon skeletons. The in situ self-assembly and phosphatisation processes enabled the firm encapsulation of CoCo-PBA-templated CoP nanocubes in a 3D graphene aerogel network (denoted as CoP@GA) [92]. As anticipated, the hierarchically porous structure and the rigid conductive substrate contributed to an outstanding initial Coulombic efficiency (ICE) of 88.6% and remarkably stable capacities (805.3 mAh g^−1^ at 200 mA g^−1^ for 200 cycles and 351.8 mAh g^−1^ at 10 A g^−1^ for 4000 cycles). These results manifest that the carbon matrix is not only a continuous conductive network to expedite the electrochemical kinetics of metal phosphide electrodes but also an elastic buffer to reduce the aggregation issue of nanoparticles.

Considering the effectiveness of carbon encapsulation, the “assembly and phosphorisation” route was adopted in the modification of multi-component/multi-metallic phosphides. Recently, Ou et al. proposed a facile synthetic route to fabricate the monodispersed electroactive CoP/Co_2_P nanoparticles in N-doped carbon matrix interlaced by CNTs (CoP/Co_2_P@N–C) beginning with the CoCo-PBA template [50]. The synergism of active components and N-doped carbon framework imparted the CoP/Co_2_P@N–C anode with impressive half cell (977.9 mAh g^−1^ at 100 mA g^−1^ for 100 cycles) and full cell (137.5 mAh g^−1^ at 100 mA g^−1^ for 50 cycles) performances. The successful operation of the LIB full cell system with LiFePO_4_ as the cathode material indicated the potential of cobalt phosphide-based composites in practical battery application under low current densities. Similarly, another composite with NiCoP nanoparticles in N-doped carbon (NiCoP@N–C) was meticulously designed by a two-step phosphorisation process, during which the pyrolysis temperature is vital to the resulting lithium storage properties [38]. When the pyrolysis temperature was 600 °C, the resulting NiCoP@N–C composite (P-NCP-NC-600) outperformed other counterparts in lithium storage due to the optimal structural properties and appropriate crystallinity degree/N content. As presented in Fig. 9e, P-NCP-NC-600 exhibited extraordinary capacity retention at elevated current densities (422.4 mAh g^−1^ at 5000 mA g^−1^) during the rate performance measurement. Moreover, theoretical studies suggested the excellent synergism of N-doped carbon and NiCoP nanoparticles and further supported the credibility of experimental results.

#### Others

**Fig. 10:**
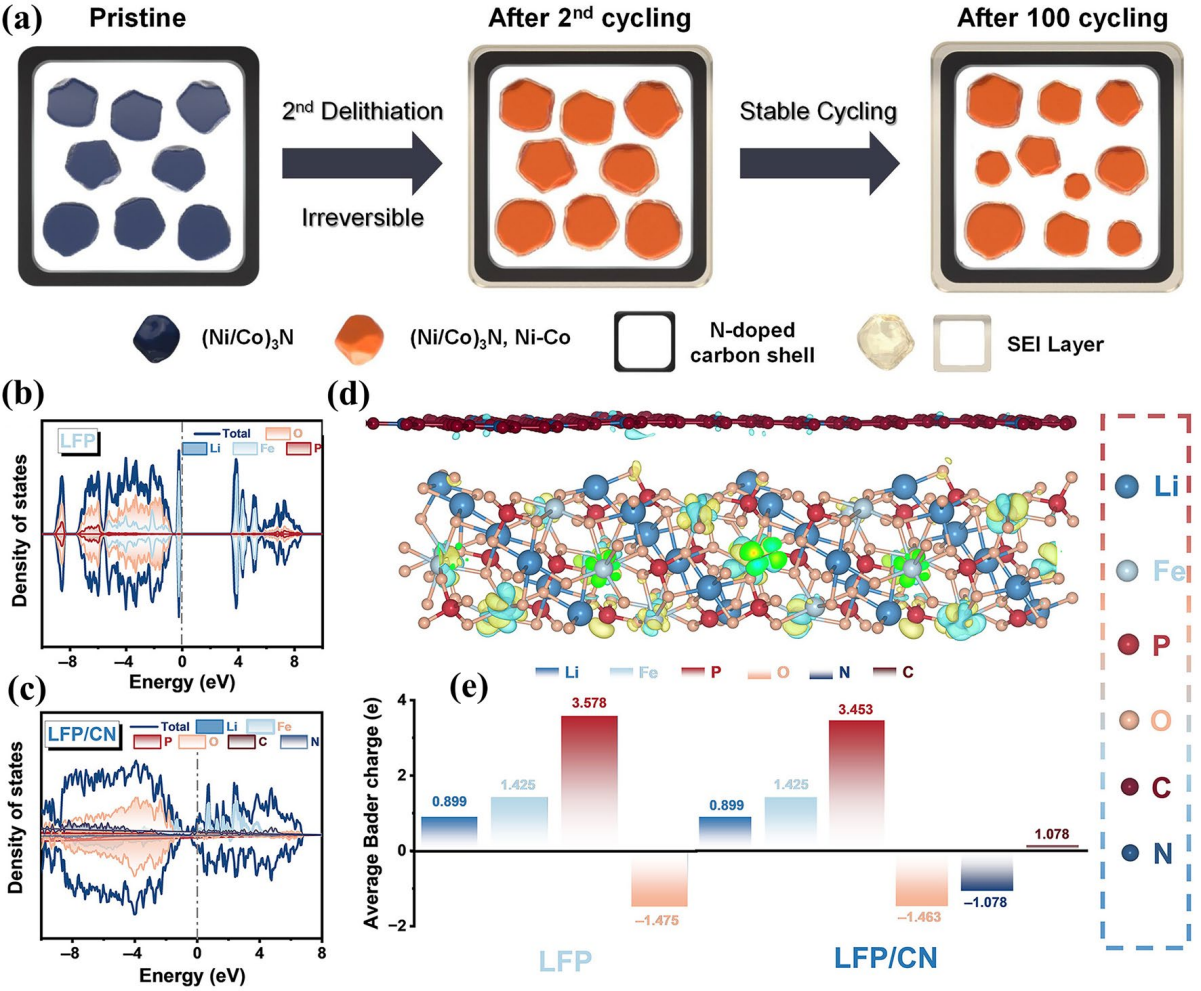
**a** Lithium storage process of (Ni/Co)_3_N MC@HC. Reproduced with permission from Ref. [73], Copyright 2021, Elsevier. The density of states (DOS) curves of **b** LFP and **c** LFP/CN; **d** Differential charge density distribution of LFP/CN; **e** Bader charge distribution of LFP and LFP/CN. Reproduced with permission from Ref. [40], Copyright 2023, Wiley–VCH

Apart from prevailing metal compounds, PBAs are also suitable templates for fabricating other LIB anodes by precise control of calcination conditions. As mentioned earlier, the Co_3_ZnC phase can be generated by the thermal decomposition of ZnCo-PBA at 600 °C in N_2_ for 2 h, whose lithium storage mechanism has not been studied thoroughly [49, 72]. Despite the unclear mechanism, Co_3_ZnC nanoparticles in carbon microspheres (Co_3_ZnC/C) derived from ZnCo-PBA delivered enticing cycling stability as the LIB anode (608 mAh g^−1^ at 100 mA g^−1^ for 300 cycles; 423 mAh g^−1^ at 1000 mA g^−1^ for 1150 cycles). The ZnO/Co_3_ZnC/N-doped carbon composite also surpassed ZnO in cyclability (1162.4 mAh g^−1^ at 200 mA g^−1^ for 300 cycles) and multiplier behaviour (473.3 mAh g^−1^ at 1000 mA g^−1^), indicating the enticing electrochemical properties provided by Co_3_ZnC. The reductive atmosphere containing N_2_ and NH_3_ for the pyrolysis of PBAs can facilitate the formation of metal nitrides with intriguing lithium storage properties. The composite with (Ni/Co)_3_N multi-core nanoparticles encapsulated in the hollow N-doped carbon shell (denoted as (Ni/Co)_3_N MC@HC) prepared by the thermolysis of NiCo-PBA@PDA delivered stable cycle life (~ 440 mAh g^−1^ at 200 mA g^−1^ for 130 cycles) compared with its counterpart without the outer carbon shell [73]. This result can be attributed to the formation of a stable solid electrolyte interface (SEI) film, the robust carbon shell without structural variation upon cycling, and the reversible conversion between metal nitride and metal phases (Fig. 10a). Considering the suitability of graphite as the LIB anode, PBA-templated porous carbon materials can realise high-efficiency lithium storage process by reversible intercalation reactions. Through a carbonnitridation pyrolysis process of the site-selective etched NiCo-PBA, a hybrid with Ni-Co nanoparticles embedded in carbon nanotubes (NCCH) was developed [85]. The NCCH hybrid anode displayed excellent reversible capacities (over 400 mAh g^−1^) comparable with conventional graphite owing to the peculiar hierarchical architecture with ample active sites. The investigation of these unusual LIB anode materials extends the possibilities for the application of PBAs in the LIB industry.

Most PBA-templated nanocomposites have been reported as advanced anode materials, but PBAs were employed as templates for intercalation-type LIB cathode material design in some cases. Co_3_[Co(CN)_6_]_2_ was reported as a feasible template for the fabrication of LiCoO_2_ nanoparticles by the high-temperature lithiation step, but the resultant product did not perform well when evaluated at the voltage range of 3.12–4.3 V (101.6 mAh g^−1^ at 1 C for l00 cycles) [79]. A similar strategy was also applied in the directional preparation of NCM materials using (Na_0.25_K_0.15_)-Ni_2.6−x_Mn_x_[Co(CN)_6_]_2_ as the precursor [80]. Attributed to the efficient phase transition during lithiation, the resulting NCM materials delivered minimal Li-Ni cation disorder (~ 1 atom%) and hence exhibited considerable cyclability (80% retention at 0.5 C after 100 cycles) and rate capability (136 mAh g^−1^ at 10 C). Therefore, PBAs are promising templates to morphologically and atomically control the resulting Li-based layered metal oxides. As another successful commercial LIB cathode, LFP has also been controllably designed by the PBA-templated route. One primary consideration is to establish conductive layers to address the inherently low conductivity of primitive LFP [40]. As elucidated by the theoretical studies (Fig. 10b–e), the successful retention of N-doped carbon from the FeFe-PB template endowed the LFP/CN composite with boosted electronic conductivity and ameliorated electronic configuration. Although N-doped carbon can unambiguously ameliorate the electrochemical properties of LFP (153.2 mAh g^−1^ at 0.5 C after 500 cycles for LFP/CN), there remains some space for improvement in working voltage and energy density, inspired by which MnFe-PBAs with various Mn/Fe ratio were utilised to accurately manipulate the Mn doping ratio of LiMn_*x*_Fe_1–*x*_PO_4_/C composites [41]. When *x* = 0.25, the LiMn_0.25_Fe_0.75_PO_4_/C cathode presented superior electrochemical performances owing to the optimal morphology, N-doped carbon coating, and the unique single-phase solid solution mechanism induced by proper Mn doping. Despite the inadequacy of studies on PBA-templated LIB cathodes, these results provide valuable insights into the directional modification of these materials.

### Sodium-Ion Batteries

The similarity of SIBs compared with LIBs imparts pristine PBAs with remarkable potential as intercalation-type sodium storage materials due to the adjustable open framework structure. Moreover, PBA-templated nanocomposites not only retain porosity but also provide additional sodium storage activities by generating electrochemically active components, which are promising SIB electrodes with exalted capacities [5]. Inspired by this, this section focuses on the correlation between sodium storage properties of PBA-templated nanocomposites and their compositional/structural features, as tabulated in Table 2.

**Table 2:** Sodium storage properties of PBA-templated nanocomposites

Electrodes	Templates	Initial DC/CC (mAh g^−1^)	Voltage (V vs. Na^+^/Na)	ICE (%)	RC/rate (mA g^−1^)	Cycle number	References
MgFe_2_O_4_	Fe_4_[Fe(CN)_6_]_3_	406/207	0.005–3.0	51	135/50	150	[99]
PCFO-NCs	CoFe-PBA	573/394	0.005–3.0	68.8	360/50	50	[97]
PCFO-NCs	CoFe-PBA	573/394	0.005–3.0	68.8	394/50	50	[98]
NiO-Co_3_O_4_@rGO	NiCo-PBA	789/281	0.01–3.0	35.6	269/100	100	[137]
NiS	NiNi-PBA	763/381	0.005–3.0	50	166/1000	100	[103]
NiS_2_@CoS_2_@C@C	NiCo-PBA	−	0.01–3.0	−	600/1000	250	[138]
Fe_7_S_8_@NC@MoS_2_	FeFe-PB	−	0.01–3.0	−	600/100	100	[100]
PFS@NC	FeFe-PB	−	0.5–3.0	−	375/5000	1000	[140]
ReS_2_/C	FeFe-PB	540/400	0.01–2.5	74	290/200	200	[141]
FeCo-NiS@NC	NiCoFe-PBA	700/578	0.01–3.0	82.6	454/100	100	[94]
Co_3_S_4_@C-N/S	CoCo-PBA	745/685	0.3–3.0	91.97	599/1000	600	[142]
Co_9_S_8_@C-N/S	CoCo-PBA	627/539	0.3–3.0	86.1	392/2000	1200	[142]
(Ni_0.5_Co_0.5_)_9_S_8_@NC	Ni_3_[Co(CN)_6_]_2_	1031/776	0.01–3.0	75	752/100	100	[36]
NiS_2_/CoS_2_@NC@C	NiCo-PBA	921/808	0.01–3.0	87.7	490/1000	170	[145]
NiCoS_4_@ReS_2_	NiCo-PBA	716/545	0.01–3.0	76.1	396/1000	500	[146]
NCS@NDDC	Ni_3_[Co(CN)_6_]_2_	654/444	0.01–2.8	68	379/500	100	[74]
MnS-FeS_2_@NSC	MnFe-PBA	673/458	0.01–3.0	68.7	501/100	800	[37]
Fe-CoS_2_/NC	FeZnCo-PBA	850/ −	0.01–3.0	−	621/1000	400	[144]
MnS-CoS_2_-NC@NC	MnCo-PBA	814/620	0.01–3.0	76.2	609/200	100	[102]
Fe_0.6_Co_0.3_Ni_0.1_S_2_@NC	FeCoNi-PBA	1850/−	0.01–3.0	−	280/10000	400	[147]
CoS_2_/FeS_2_@CNFs	CoFe-PBA	800/514	0.01–3.0	64.3	543/500	150	[57]
Ni_0.67_Fe_0.33_Se_2_/NC	NiFe-PBA	458/355	0.5–3.0	77.5	375/10000	10,000	[148]
ZnSe@CoSe_2_	Zn_3_[Co(CN)_6_]_2_	625/613	0.01–3.0	98	615/1000	500	[149]
Cu_2_Se@CoSe_2_	Cu_3_[Co(CN)_6_]_2_	568/445	0.01–3.0	78	460/1000	500	[149]
ZnSe/FeSe	Zn_3_[Fe(CN)_6_]_2_	669/493	0.01–3.0	73.7	549/100	70	[53]
ZnSe-Fe_3_Se_4_@NC	Zn_3_[Fe(CN)_6_]_2_	906/605	0.005–3.0	66.8	368/100	60	[75]
Ni_0.6_Fe_0.4_Se_2_@NC	Ni_3_[Fe(CN)_6_]_2_	503/449	0.5–3.0	94	372/5000	2000	[150]
3DOM-MnFeSe_x_@C	MnFe-PBA	539/450	0.01–3.0	83.5	460/200	70	[151]
Cu-CoSe_2_ NFCs	Co_3_[Co(CN)_6_]_2_	470/ −	0.01–3.0	−	423/100	100	[63]
FeSe_2_/NC@G	Fe_4_[Fe(CN)_6_]_3_	799/529	0.01–3.0	66	477/100	50	[152]
N/(Ni, Co)Se_2_ NFCs@RGOA	NiCo-PBA	636/482	0.01–3.0	76	395/100	200	[58]
FePNC	FeFe-PB	740/506	0.01–2.5	68.4	275/200	200	[104]
rGO@CoP@FeP	FeFe-PB	968/551	0.01–3.0	56.9	456/100	200	[77]
T-FeP	T-PB	442/342	0.01–3.0	77.2	391/100	100	[78]
FeP@C	PB	647/346	0.01–3.0	53.4	368/100	100	[153]
Cu-Fe–P/TiN	K_2_CuFe(CN)_6_	355/245	0.01–3.0	69	120/50	100	[105]
CoSe_2_/Fe_3_C	Co_3_[Fe(CN)_6_]_2_	427/411	0.01–3.0	96.4	412/10000	2000	[154]

DC/CC: discharge/charge capacity, ICE: initial Coulombic efficiency, RC: reversible capacity

#### Metal Oxides

Metal oxides have been well reported in lithium storage, which can also deliver enticing sodium storage performances based on similar conversion/alloying reactions [139]. To mitigate the structural deterioration during repetitive Na^+^ insertion/extraction, structural engineering is crucial for metal oxide-based SIB anodes, which can be facilely implemented by employing PBAs as self-sacrificial templates. The thermal treatment in air usually results in metal oxides with hollow structures, before which metal species are adjustable by “wet chemistry” approaches. For instance, the fabrication of MgFe_2_O_4_ microboxes began with the preparation of Fe_4_[Fe(CN)_6_]_3_ followed by the ion-exchange reaction and the subsequent thermolysis, yielding a considerable initial capacity of 406 mAh g^−1^ at 50 mA g^−1^ but a low capacity retention (33% retention after 150 cycles) [99]. Similarly, porous CoFe_2_O_4_ nanocubes (PCFO-NCs) derived from CoFe-PBA presented remarkable reversible capacities of 360 mAh g^−1^ at 50 mA g^−1^ after 50 cycles and 152.6 mAh g^−1^ at 2500 mA g^−1^ after 500 cycles in SIB half cells [97]. This embodies the significance of the particle size for metal oxides in sodium storage, whereas nanocubes with smaller particle sizes can effectively shorten the distance for Na^+^ diffusion. The selection of binders also shows a significant impact on the resulting sodium storage performance of PCFO-NCs, which was experimentally demonstrated by Zhang and coworkers [98]. Notably, the carboxyl methyl cellulose (CMC) binder boosted sodium diffusivity, ameliorated the electrode/electrolyte interfacial property, and hence distinctly elevated the cycling stability with negligible capacity attenuation at 50 mA g^−1^ after 50 cycles, thereby elucidating the feasibility of the CMC binder in SIB systems. Despite the plentiful void space affording Na^+^ insertion/extraction, the subpar conductivity and structural vulnerability are challenging for bare metal oxide SIB anode materials.

To reconcile the above issues, the design of metal oxide-based carbonaceous materials has been attempted for prolonged cycle life. Recently, the rGO-encapsulated NiO-Co_3_O_4_ open-ended hollow nanocube (NiO-Co_3_O_4_@rGO) composite was reported as an advanced SIB anode [137]. Bestowed by the robust conductive network and the peculiar hollow architecture, the NiO-Co_3_O_4_@rGO composite delivered a stable sodium storage capacity of 269.2 mAh g^−1^ at 100 mA g^−1^ for 100 cycles due to the mitigated volume change. However, a disappointing ICE value of < 40% was achieved, which remains an inevitable issue for nanostructural metal oxide carbonaceous composites and requires further modification.

#### Metal Chalcogenides

In contrast with the lacklustre progress of PBA-templated metal oxides in sodium storage, metal chalcogenides (metal sulphides and metal selenides) are deemed as promising candidates as SIB anodes due to the impressive theoretical sodium storage capacity and superior electrical conductivity [103], whose directional synthesis has been widely reported by the PBA-derived routes. Inspired by the enticing lithium storage properties of PBA-derived metal sulphides, in recent years, PBA-templated metal sulphides and their carbon composites, viz. NiNi-PBA-derived NiS [103], NiS_2_@CoS_2_@C@C derived from NiCo-PBA@PDA [138], PB-templated Fe_7_S_8_@NC@MoS_2_ heterostructures [100], and trimetallic PBA-derived FeCo-NiS@NC [94], were extensively investigated in sodium storage, resulting in impressive reversible capacities (usually over 400 mAh g^−1^). Monometallic metal sulphide carbon composites are accessible by the thermal sulphuration of monometallic PBAs, during which the introduction/removal of components for desirable products can be flexibly executed. By PPy wrapping and sulphuration processes of PB nanocubes, a york–shell composite with FeS_2_ nanocages confined in N-doped carbon (denoted as PFS@NC) was obtained [140]. The exogenous carbon shell not only established a robust conductive network but also trapped the active FeS_2_, thereby leading to intriguing cyclability (92% capacity retention at 5 A g^−1^ for 1000 cycles). Notably, Fe species in the PB template could be removed by in situ carbonisation and HCl leaching, after which ReS_2_/C nanostructures with regular shapes were acquired via a hydrothermal sulphurisation process [141]. The vertical growth of ReS_2_ on the PBA-derived porous carbon skeleton caused a large surface area (~ 120 m^2^ g^−1^), well-developed porosity, and plentiful reactive sites, thereby enduing the ReS_2_/C nanocomposite with considerable reversible capacity (365 mAh g^−1^ at 100 mA g^−1^) and prolonged cycle life (over 600 cycles at an elevated current density of 2 A g^−1^). Other than the typical cubic architecture, the controlled crystal growth can significantly increase the morphological diversity of the resulting products. A tremella-like microflower structure was available by the joint effect of trisodium citrate dihydrate and K_3_[Co(CN)_6_], which resulted in the formation of Co_3_S_4_ in N, S-co-doped carbon (Co_3_S_4_@C-N/S) and Co_9_S_8_ in N, S-co-doped carbon (Co_9_S_8_@C-N/S) after sulphurisation with TAA and S powders as S sources, respectively [142]. As anticipated, these cobalt sulphide carbon composites exerted excellent cyclability (~ 600 mAh g^−1^ at 1000 mA g^−1^ for 600 cycles for Co_3_S_4_@C-N/S; ~ 390 mAh g^−1^ at 2000 mA g^−1^ for 1200 cycles for Co_9_S_8_@C-N/S) and remarkable ICE values (91.97% and 86.1% for Co_3_S_4_@C-N/S and Co_9_S_8_@C-N/S, respectively).

**Fig. 11:**
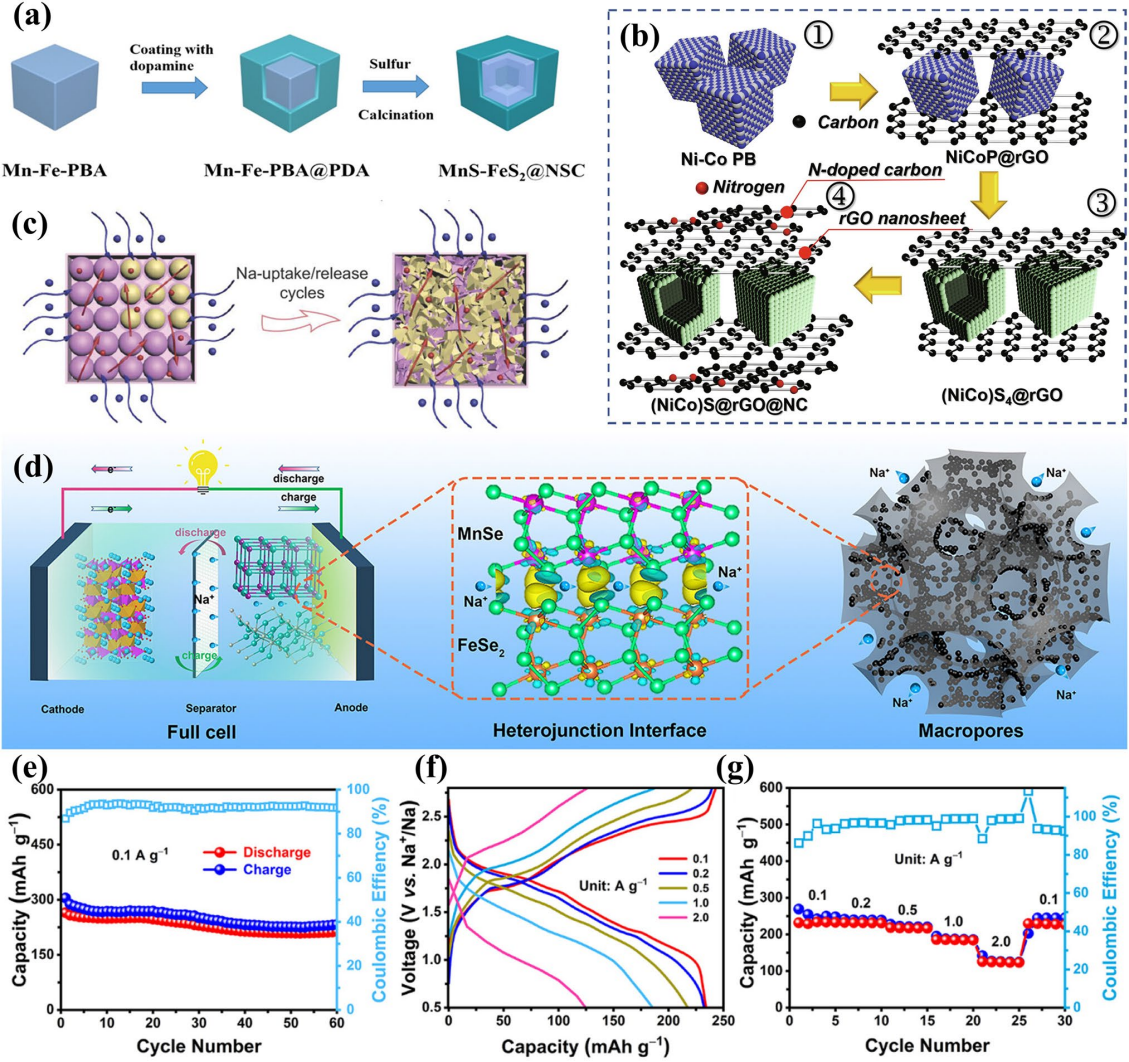
**a** Two-step fabrication of MnS-FeS_2_@NSC. Reproduced with permission from Ref. [37], Copyright 2020, American Chemical Society. **b** Synthetic process of (NiCo)S@rGO@NC. Reproduced with permission from Ref. [101], Copyright 2020, Elsevier. **c** Na^+^/electron transport pathway of NCS@NDDC after repetitive Na^+^ uptake/release. Reproduced with permission from Ref. [74], Copyright 2018, Wiley–VCH. **d** SIB full cell assembly, **e** cycling performance, **f** galvanostatic charge/discharge (GCD) curves, and **g** rate capability in the SIB full cell for 3DOM-MnFeSe_x_@C. Reproduced with permission from Ref. [151], Copyright 2023, American Chemical Society

The above results substantiate the suitability of monometallic metal sulphides (i.e. iron sulphides, rhenium sulphides, cobalt sulphides) as advanced sodium storage materials and hence prompt the targeted fabrication of metal sulphide-based nanocomposites with binary/multiple metal centres beginning with PBA templates for strengthened synergism. The multi-step pyrolysis after the self-polymerisation process of RF on Ni_3_[Co(CN)_6_]_2_ enabled the synchronous morphological engineering and N-doped carbon coating of NiCo bimetallic sulphide hollow nanocubes (denoted as (Ni_0.5_Co_0.5_)_9_S_8_@NC), enabling remarkable cycling performance (752 mAh g^−1^ at 100 mA g^−1^ for 100 rounds) due to the excellent synergistic effect of metal sulphide and carbon layer [36]. Similarly, N, S-co-doped carbon coating of MnS-FeS_2_ heterostructures (denoted as MnS-FeS_2_@NSC) could be realised by PDA coating and sulphuration with sulphur powders beginning with MnFe-PBA (Fig. 11a), resulting in extraordinary long-term cyclability (134 mAh g^−1^) at an elevated current density of 4 A g^−1^ due to the predominant pseudocapacitive dominating process [37]. Another work also reported the substantial reversible capacity (486.6 mAh g^−1^ at 100 mA g^−1^) and satisfying cycling stability (230 mAh g^−1^ at even 10 A g^−1^ over 900 cycles) of Fe_0.4_Co_0.6_S_2_@NC synthesised via the PDA coating step of CoFe-PBA and succedent sulphuration [143]. Conspicuously, the heteroatom-doped carbon layer is kinetically propitious to Na^+^/electron transportation and can mitigate the mechanical stress upon long-term cycling. The incorporation and magnification of active sites should be taken into account when designing and applying these materials, as exemplified by the enticing sodium storage behaviour of N-doped carbon-confined Fe-doped CoS_2_ nanoparticles (Fe-CoS_2_/NC) templated by the FeZnCo-PBA [144]. The evaporation of Zn species creating vast defects, the perfectly retained N-doped carbon framework, and the additional activities induced by Fe^3+^ doping profoundly endowed the Fe-CoS_2_/NC with remarkable half cell (621 mAh g^−1^ at 1 A g^−1^ for 400 rounds; 493 mAh g^−1^ at 5 A g^−1^) and full cell (313 mAh g^−1^ at 500 mA g^−1^ for 60 cycles with a retention rate of 83.9%) performances in SIBs.

Taking the fabulous advantages of carbon layers into account, the double carbon tactic has been employed in metal sulphide-based SIB anode modification for stabilised inner active materials and expedited charge transfer. For example, the double N-doped carbon coating of MnS-CoS_2_ heterostructures (abbreviated as MnS-CoS_2_-NC@NC) was implemented by typical PDA coating and solid-state sulphuration protocols [102]. Incredible cycling stability (560.5 mAh g^−1^ at 5 A g^−1^ after 1100 cycles) was exerted by the MnS-CoS_2_-NC@NC in SIB half cells, while an exciting result (436.7 mAh g^−1^ at 1 A g^−1^ after 900 cycles) was presented when evaluating the MnS-CoS_2_-NC@NC anode in the SIB full cell system with Na_3_V_2_(PO_4_)_3_ as the cathode, thereby manifesting the practicability of double carbon-modified materials. A similar conclusion was entailed in another study related to the sodium storage properties of NiCo-PBA-derived NiS_2_/CoS_2_ embedded N-doped carbon nanocubes in RF-derived carbon shell (nominated as NiS_2_/CoS_2_@NC@C) [145]. The noticeable cyclability and ICE of this composite could be assigned to the structure rigidity induced by hierarchical carbon matrices, unimpeded ion/electron pathways, thorough electrolyte/active material contact, and electron redistribution triggered by the heterogeneous interface.

Despite the undisputed merits of the carbon encapsulation strategy, the resulting products are prone to present dissatisfactory volumetric/mass energy density, which can be overcome by enhancing the volume/mass ratio of electrochemically active entities. One viable strategy is to increase active centres in metal sulphide carbonaceous materials for maximised sodium storage contribution. ReS_2_ can provide exceptional sodium storage capability and hence can increase sodium active sites. Motivated by this, a hierarchical nanomaterial with ReS_2_ anchored on NiCoS_4_ carbonaceous nanocubes (NiCoS_4_@ReS_2_) was fabricated by a two-step sulphuration route [146], where ReS_2_ exerted additional electrochemical activities. By coupling the inner void space and outer ReS_2_ nanosheets, the velocity of ion/electron transfer could be enhanced, thereby demonstrating stable reversible capacities (408 mAh g^−1^ at 0.5 A g^−1^ for 150 cycles; 396 mAh g^−1^ at 1 A g^−1^ for 500 cycles). Another recent study illustrated trimetallic sulphide nanoboxes coated by N-doped carbon (Fe_0.6_Co_0.3_Ni_0.1_S_2_@NC) manufactured by in situ sulphuration of PDA-wrapped FeCoNi-PBA with splendid sodium storage capacities (1000 and 390 mAh g^−1^ at 0.1 and 10 A g^−1^, respectively) [147]. This can be attributed to the conformal carbon layer and the complementary behaviour of multiple active sites, thereby experimentally validating the good synergism in trimetallic sulphide SIB anodes. Another emerging methodology to pursue higher energy density is the establishment of “binder-free” electrodes with conductive substrates. Starting from NiCo-PBA, a conceptual methodology was envisioned to implement rGO encapsulation of NiCo sulphide nanocubes, after which N-doped carbon coating was realised by proper post-treatment (denoted as (NiCo)S@rGO@NC), as illuminated in Fig. 11b [101]. The rGO and N-doped carbon matrix simultaneously extend the transmission aisles for electrons, thereby promoting capacity retention at high current densities (413 mAh g^−1^ at 10 A g^−1^). The electrospinning protocol with PAN was also reported in the fabrication of PBA-derived hollow CoS_2_/FeS_2_ nanoparticles confined in N, S-co-doped carbon nanofibers (H-CoS_2_/FeS_2_@CNFs) via two-step in situ thermal conversion [57]. The heteroatom-doped CNFs warranted the velocity of charge transfer and inhibited particle aggregation, resulting in remarkable sodium storage capability (323.7 mAh g^−1^ at 5 A g^−1^ over 1500 cycles during the half cell measurement and 240.6 mAh g^−1^ at 0.5 A g^−1^ over 200 cycles during the full cell measurement using Na_3_V_2_(PO_4_)_3_ as the cathode) and further inspiring the rational design of bimetallic sulphide SIB anodes by spatial confinement and heterostructural engineering.

The above methodologies have also garnered broad attention in the development of metal selenide-based SIB anodes considering the similarity of metal chalcogenides in sodium storage. To our best knowledge, the robust N-doped carbon shell can form a continuous conductive network and simultaneously minimise the mechanical strain during cycling, thereby leading to conspicuous stability in both SIB half cells and full cells. Taking the composite with Ni_1.8_Co_1.2_Se_4_ nanoparticles in N-doped dual carbon (NCS@NDDC) derived from NiCo-PBA as an example, incredible rate capability (153 mAh g^−1^ at even 50 A g^−1^) and cyclability (379.3 mAh g^−1^ at 500 mA g^−1^ for 100 cycles) were achieved in SIB half cells, while an extraordinary energy density of 227 Wh kg^−1^ was displayed during the full cell measurement coupling with the Na_3_V_2_(PO_4_)_2_O_2_F cathode [74]. As exhibited in Fig. 11c, the structural deterioration was productively accommodated by the carbon shell without destruction, during which fast electron/ion transport could be maintained upon cycling. The outstanding sodium storage property of N-doped carbon-coated binary metal selenides prompted another attempt to construct the SIB full cell system with NiFe-PBA as cathode and its derived Ni_0.67_Fe_0.33_Se_2_ in N-doped carbon (Ni_0.67_Fe_0.33_Se_2_/NC) [148]. Remarkably, the as-assembled full cell displayed a reversible capacity of 302.2 mAh g^−1^ at 1 A g^−1^ owing to the synergistic effect of Ni and Fe metal centres triggering more lattice defects and hence accelerating the electrochemical kinetics. It is widely acknowledged that the resulting morphology of metal selenide carbon composite can be determined by the selection of metal nodes. The facile bottom-up fabrication route enabled the formation of ZnSe@CoSe_2_ microspheres and Cu_2_Se@CoSe_2_ nanocubes using Zn_3_[Co(CN)_6_]_2_ or Cu_3_[Co(CN)_6_]_2_ as precursors, respectively [149]. These PBA-templated selenide composites as SIB anodes delivered satisfying cyclability (525 and 423 mAh g^−1^ at 5 A g^−1^ after 500 cycles for ZnSe@CoSe_2_ and Cu_2_Se@CoSe_2_, respectively) due to the well-inherited primitive structures and hollow porous architectures. In another report, ZnSe/FeSe nanospheres with carbon coating were developed beginning with ZnFe-PBA nanospheres [53]. The ZnSe/FeSe heterostructure could trigger disordered atom arrangements and hence modulate the electronic/interfacial characteristics, thereby contributing to the expedited sodium storage kinetics and outstanding capacities (524.6 and 417.9 mAh g^−1^ at 100 and 2000 mA g^−1^, respectively). The hybrid mechanism (alloying and conversion) of ZnSe-based heterostructures endows them with considerable reversible capacities in SIBs.

Considering the significance of carbon layers in structural consolidation and conductivity amelioration, the hierarchical carbon modification tactic was also applicable in the design of metal selenide-based SIB anodes. As mentioned earlier, the PDA coating strategy can successfully integrate endogenous and exogenous carbon matrices to extend the conductive network. Ascribed to the fast velocity of Na^+^ transfer endowed by functional Fe–N–C bonds and the prominent pseudocapacitive contribution imparted by the hierarchical structure, the hierarchically N-doped carbon-wrapped Ni_0.6_Fe_0.4_Se_2_ (termed as Ni_0.6_Fe_0.4_Se_2_@NC) exhibited superiority in sodium storage behaviours (449.3 and 289.5 mAh g^−1^ at 0.2 and 10 A g^−1^, respectively) [150]. Motivated by the enticing sodium storage properties of heterostructures, Zhang et al. employed a hierarchical composite with ZnSe-Fe_3_Se_4_ heterostructures in N-doped carbon (ZnSe-Fe_3_Se_4_@NC) derived from PDA-coated ZnFe-PBA as an advanced SIB anode [75]. The mitigated volume variation, well-maintained structural integrity, and homogeneity of heterostructures endowed the ZnSe-Fe_3_Se_4_@NC composite with a stable sodium storage capacity of 368 mAh g^−1^ at 100 mA g^−1^ over 60 cycles. The balanced merits of heterostructures and hierarchical architectures show promising potential for advanced sodium storage due to the plentiful active sites and considerable energy density. To develop the hierarchical porous structure, the polystyrene sphere (PS) was utilised as a template for controllable PBA growth to attain the 3D macroporous carbon-modified MnFe selenide (nominated as 3DOM-MnFeSe_x_@C) via chemical soaking, tetrahydrofuran (THF) etching, and in situ selenisation procedures [151]. Coupled with the hierarchical macroporosity and heterojunction interface, enhanced mass transfer efficiency and faster Na^+^ adsorption/conversion could be attained, thereby promoting the excellent sodium properties of 3DOM-MnFeSe_x_@C (Fig. 11d). Aside from the remarkable SIB half cell performance (277.6 mAh g^−1^ at 10 A g^−1^), the impressive performance in the SIB full cell (Fig. 11e–g) of 234.1 and 124.6 mAh g^−1^ at 0.1 and 2 A g^−1^, respectively, with Na_3_V_2_(PO_4_)_3_@C as the cathode further elucidated the practicability of this composite anode material. These results offer more theoretical and experimental insights into the structural and compositional engineering of heterostructural metal selenide-based nanocomposites with PBA templates. More impressively, numerous successful cases in full cell assembly using PBA-templated metal chalcogenides as anode materials reveal the potential possibilities for the practical SIB application of metal chalcogenides with the PBA-templated strategy.

The enhancement of energy density is also a key factor in the practical use of PBA-templated metal selenide-based SIB anodes, which requires the pursuit of a higher proportion of active components in these electrodes. Although PBA-derived selenides were prevailingly prepared by solid-state thermolysis with the retention of carbon matrices, there remain some attempts to fabricate metal selenides by “wet chemistry” approaches for reinforced environmental benefits and maximised effective sodium storage volume. Starting from CoCo-PBA, ammonia etching was executed to generate a hollow cubic architecture, after which ion-exchange reactions enabled the formation of Cu-doped CoSe_2_ nanoframe cubes (Cu-CoSe_2_ NFCs) [63]. Owing to the hollow structure facilitating ion diffusivity and the synergism between different metal centres, the Cu-doped sample surpassed its counterpart without Cu doping in rate capabilities (470 and 387 mAh g^−1^ at 0.1 and 2 A g^−1^, respectively). Similar to metal sulphides, conductive substrates (i.e. graphene [152], reduced graphene oxide aerogel [58]) were introduced by controlled crystallisation of PBAs to establish “binder-free” selenide-based electrodes with strengthened mechanical robustness and enhanced electrical conductivity. Among these, PB-derived FeSe_2_ nanoparticles in N-doped carbon anchored on the graphene layer (abbreviated as FeSe_2_/NC@G) and NiCo-PBA-derived N-doped (Ni, Co)Se_2_ nano-framework cubes on reduced graphene oxide aerogel (abbreviated as N/(Ni, Co)Se_2_ NFCs@RGOA) performed satisfactorily when employed as SIB anodes, particularly at elevated current density (331 mAh g^−1^ at 5 A g^−1^ for FeSe_2_/NC@G; 389 mAh g^−1^ at 2 A g^−1^ for N/(Ni, Co)Se_2_ NFCs@RGOA), which can be assigned to thin and sturdy conductive networks with excellent chemical affinity.

#### Metal Phosphides

**Fig. 12:**
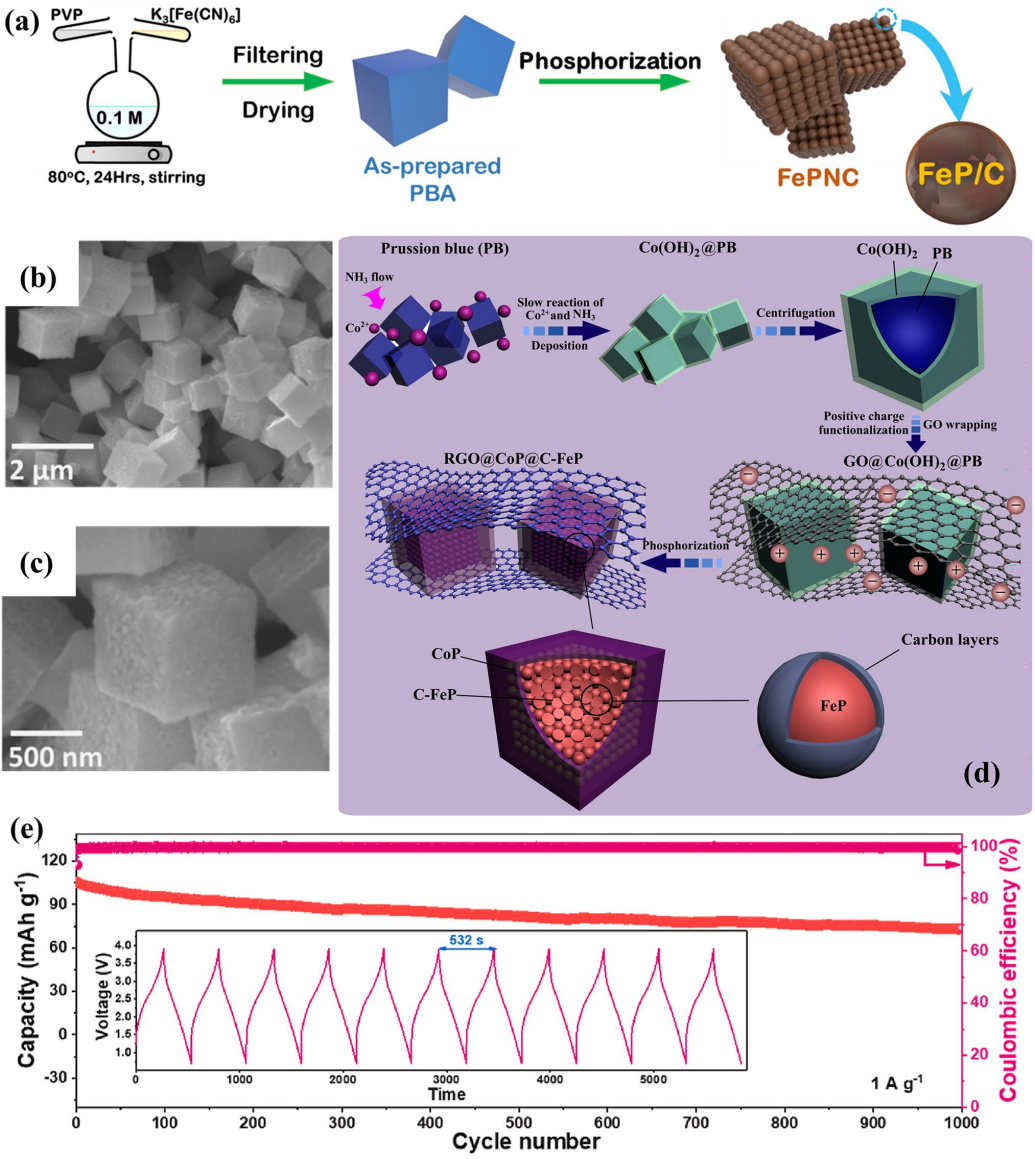
**a** Fabrication and **b, c** SEM micrographs of FePNC. Reproduced with permission from Ref. [104], Copyright 2018, Elsevier. **d** Preparation of the RGO@CoP@FeP composite. Reproduced with permission from Ref. [77], Copyright 2017, Elsevier. **e** Long-term cyclability of T-PB||T-FeP SIB full cell at 1 A g^−1^. Reproduced with permission from Ref. [78], Copyright 2023, Elsevier

Relying on conversion reactions, metal phosphides feature low Na^+^ intercalation potential and substantial theoretical capacity and hence have been regarded as promising SIB anodes, among which iron phosphides and their composites have garnered particular interest due to their considerable theoretical capacities (> 900 mAh g^−1^) and environmental friendliness. Through a chemical vapour deposition process, the direct phosphorisation of cubic PB (500–700 nm) could be implemented to acquire FeP nanoparticles in N-doped carbon matrix (FePNC) [104], as schematically illustrated in Fig. 12a. The porous nanocubic structure was perfectly retained during this synthetic process (Fig. 12b, c), thereby resulting in remarkable sodium storage performance (410.6 and 122.8 mAh g^−1^ at 0.1 and 1 A g^−1^, respectively). Hampered by the structural frangibility and sluggish electron/ion transportation of the FeP electrode, the capacity retention rate at elevated current densities is insufficient, but these issues can be further accommodated by the incorporation of other active entities and robust conductive networks. As portrayed in Fig. 12d, the core–shell porous FeP@CoP interconnected by rGO (rGO@CoP@FeP) was synthesised by three steps: chemical deposition, electrostatic interaction, and low-temperature phosphorisation [77]. The core–shell structure with sufficient cushion space, the synergistic effect between various active components, and the interconnected conductive highways facilitated the sodium storage capacity at elevated current densities (341.2 mAh g^−1^ at 2 A g^−1^), significantly outperforming the counterparts without rGO wrapping/CoP coating. Another work attempted the in situ growth of CuFe-PBA on the Ti net (TiN) to prepare the Cu-Fe–P/TiN composites containing Cu_3_P and Fe_2_P phases [105]. Despite the superior electrochemical properties of Cu-Fe–P/TiN compared with Cu_3_P/TiN owing to the synergistic effect, the dissatisfactory reversible capacity (< 200 mAh g^−1^ at 50 mA g^−1^) lags far behind the requirement in practice. Henceforth, the rational selection and optimisation of conductive substrates ought to be further considered, which is a momentous issue for the design of “binder-free” SIB electrodes.

Based on the encouraging results of PBA-templated metal phosphide-based nanocomposites in SIB half cells, further assembly and investigation of SIB full cells have been attempted. Since the primitive PB shows considerable potential in sodium storage, a tubular PB cathode (T-PB) was hydrothermally fabricated and its derived tubular FeP anode (T-FeP) was fabricated by polymerisation and phosphorisation procedures, based on which a SIB full cell system was designed [78]. The as-assembled full cell demonstrated an impressive cycling lifespan of 102 mAh g^−1^ at 0.1 A g^−1^ after 100 cycles and 69.5% capacity retention rate at 1 A g^−1^ (Fig. 12e). Similarly, another full cell system with the cubic PB as the cathode and its derived core–shell FeP@C as the anode was constructed [153]. The FeP@C composite manifested a distinguished rate capability of 111.1 mAh g^−1^ at 20 A g^−1^ with a fabulous capacity recovery (343.3 mAh g^−1^) when the current density bounced back to 0.05 A g^−1^. Coupled with the prominent cyclability (99.7% retention after 2000 cycles) of the PB cathode, the SIB full cell exerted a reversible capacity of 108.3 mAh g^−1^ and outstanding durability (88.4 mAh g^−1^ at 0.1 A g^−1^ for 200 cycles). The construction of SIB full cells successfully substantiates the potential of metal phosphides (i.e. iron phosphides, cobalt phosphides, nickel phosphides) for practical SIB systems with PBAs or sodium metal oxides as cathodes [55]. Excitingly, enlightened by the above results, these phosphides can perform satisfactorily (over 100 mAh g^−1^) when matched with suitable SIB cathodes and are promising candidates for next-generation SIBs.

#### Others

Despite the distinguished sodium storage capacities of PBA-templated metal oxides, metal chalcogenides, and metal phosphides, most of these materials suffer from low ICE values (< 80%), thereby giving impetus to the exploration of other SIB anode materials. Metal carbides show specific lithium storage capacities as LIB anodes, motivated by which some researchers attempted to apply them in SIBs. The pyrolysis of CoFe-PBA under a specific condition (800 °C for 2 h in Ar) could facilitate the formation of Fe_3_C and Co phases, after which Co was selenised to CoSe_2_ to obtain CoSe_2_/Fe_3_C composite [154]. Impressively, a favourable SEI film could be formed in the ether-based electrolyte, which caused an extraordinary ICE of 96.4%, observably outperforming most PBA-templated conversion-type SIB anodes. Attributed to the robust and thin SEI film with a high Young’s modulus upon cycling, accelerated Na^+^ diffusion and stable reversible capacity (412.4 mAh g^−1^ at 10 A g^−1^ after 2000 cycles) could be accessed. Theoretical results also suggested the preferable Na salt decomposition in the ether-based electrolyte and further unravelled the rationality of experimental results. This case emphasises the significance of SEI formation and the synchronous optimisation of electrodes and electrolytes in SIBs.

### Potassium-Ion Batteries

As a cost-effective option, PIBs are expected to be ideal alternatives for LIBs due to the inexhaustibility of K and the higher operating voltage compared with other AIBs [106]. The rate capability of PIBs is principally determined by the mobility and reversible insertion/extraction of K^+^, which requires electrode materials with 3D open framework structures comprising diffusion aisles for accommodation of K^+^ ions with a larger size. In this context, PBAs have shown preferable properties for potassium storage due to the 3D channels, whose derivatives also performed well in PIBs due to the additional potassium storage sites and the retention of porous textures, as summarised in Table 3.

**Table 3:** Potassium storage properties of PBA-templated nanocomposites

Electrodes	Templates	Initial DC/CC (mAh g^−1^)	Voltage (V vs. K^+^/K)	ICE (%)	RC/rate (mA g^−1^)	Cycle number	References
Co_3_O_4_@C@MoS_2_	Co_3_[Co(CN)_6_]_2_	505/394	0.011–3	78	226/500	500	[155]
ZnO/ZnFe_2_O_4_	Zn_3_[Fe(CN)_6_]_2_	459/239	0.01–3.0	52	217/100	400	[107]
FeS_2_@rGO	PB	563/333	0.01–3.0	59	264/50	50	[108]
Mn-Fe-Se/CNTs	MnFe-PBA	1050/351	0.0–3.0	33.4	141/50	70	[110]
Ni–Fe-S-CNT	NiFe-PBA	1212/546	0.0–3.0	45	181/100	100	[109]
CoSe_2_/NiSe_2_@NC-CNTs	CoNi-PBA	1108/326	0.01–3.0	29	134/100	100	[111]
NCS@NC	Ni_3_[Co(CN)_6_]_2_	784/538	0.01–3.0	68.7	638/100	100	[156]
Ni-Co-S@rGO	NiCo-PBA	1021/705	0.1–3.0	69	765/200	100	[114]
CFS//g@NC	CoFe-PBA	622/436	0.01–3.0	70.2	195/500	2500	[112]
Ni–Fe-P/NC	FeFe-NiFe-PBA	725/303	0.01–3.0	41.8	287/100	500	[39]
3DG/FeP	PB	–	0.01–3.0	–	327/100	100	[113]
Co-NC	Zn_3_[Co(CN)_6_]_2_	1060/276	0.01–3.0	26.1	209/100	300	[62]
K_0.6_CoO_2−x_N_x_	Co_3_[Co(CN)_6_]_2_	86/70	1.5–4.2	81.7	66/50	400	[81]
KMFON	Mn_2_[Fe(CN)_6_]	–	1.5–4.0	–	52/100	300	[82]

DC/CC: discharge/charge capacity, ICE: initial Coulombic efficiency, RC: reversible capacity

#### Metal Oxides

PBA-templated metal oxides possess enchanting lithium storage properties, but limited successes in SIBs and PIBs have been attained owing to the intensified structural instability triggered by the magnified radii of the ionic charge carriers. The severe structural destruction and potential hysteresis of metal oxides during potassium storage promote further compositional and morphological optimisation with PBA templates. For instance, taking the enriched open channels attenuating K^+^ diffusion barriers into account, the hydrothermal growth of MoS_2_ on the CoCo-PBA@RF-derived Co_3_O_4_@C composite was conducted to construct the composite with MoS_2_ embedded in the carbon framework containing hollow Co_3_O_4_ nanoparticles (denoted as Co_3_O_4_@C@MoS_2_) [155]. The joint functionalities of Co_3_O_4_ and MoS_2_ assured the fast kinetics of multi-electron redox reactions, while the carbon matrix enhanced the overall conductivity. Other than the synergism of individual components, the structural texture with affluent interstitial space and enlarged surface area efficaciously reconcile the volume fluctuation and ameliorate electrode/electrolyte contact. Enlightened by the theoretical investigation, the optimised electronic and interfacial configuration endued the Co_3_O_4_@C@MoS_2_ composite with an eminent reversible capacity of 256 mAh g^−1^ and a distinguished capacity retention of 88.3% (500 cycles; 500 mA g^−1^). Motivated by the excellence of core–shell nanostructures in lithium storage, ZnO/ZnFe_2_O_4_ core–shell nanospheres derived from ZnFe-PBA were reported as a promising PIB anode with a stable reversible capacity (217 mAh g^−1^ at 100 mA g^−1^ over 400 cycles) [107]. It is widely admitted that the core–shell structure can offer void space to accommodate the volume expansion, which was embodied by the optimisation of thermolysis conditions based on the Kirkendall effect and further unravelled by the electrochemical measurements of this work. Noticeably, the synergistic effect of ZnO and ZnFe_2_O_4_ and the hybrid mechanism (alloying and conversion) pronouncedly enhanced the overall potassium storage capacity, while the carbon-free heterostructure is conducive to a higher volumetric energy density. The capacity retention (< 100 mAh g^−1^) at exalted current densities of PBA-templated metal oxide-based nanocomposites in potassium storage remains inadequate for commercial use and requires further improvement.

#### Metal Chalcogenides

**Fig. 13:**
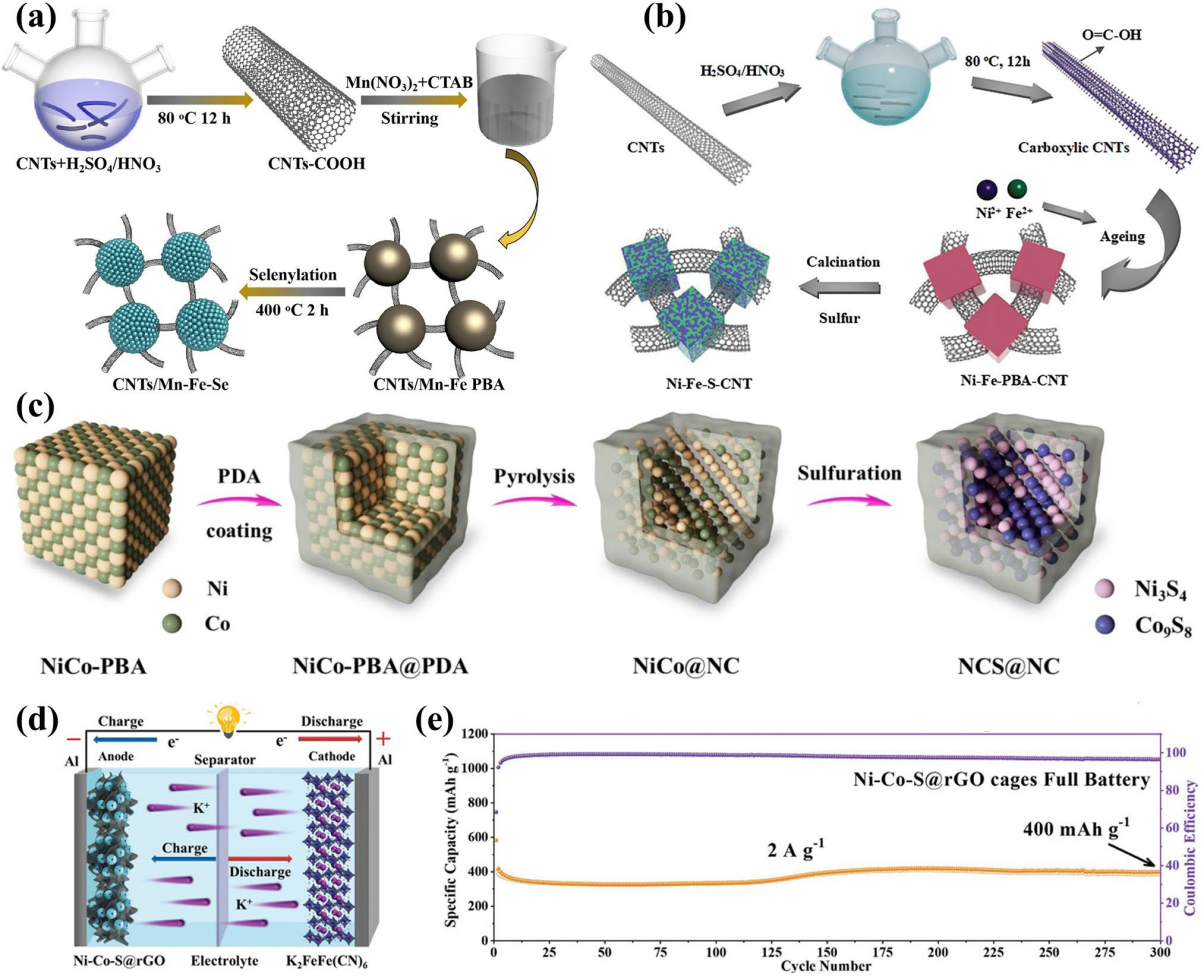
**a** Schematic illustration for the fabrication of Mn-FeSe/CNTs. Reproduced with permission from Ref. [110], Copyright 2020, Elsevier. **b** Synthesis of Ni–Fe-S-CNT. Reproduced with permission from Ref. [109], Copyright 2020, Wiley–VCH. **c** Two-step thermal fabrication of NCS@NC. Reproduced with permission from Ref. [156], Copyright 2022, Elsevier. **d** Assembly and **e** long-term cycling durability of the Ni-Co-S@rGO||K_2_FeFe(CN)_6_ PIB full cell. Reproduced with permission of Ref. [114], Copyright 2022, Wiley–VCH

Following the outstanding sodium storage properties of PBA-templated metal chalcogenides (metal sulphides and metal selenides), their potassium storage performances are also evaluated in this section. Mechanical robustness is a prime consideration for tolerance of the larger size of potassium ions, which can be probably achieved by the adoption of conductive substrates. The two-step thermal treatment of the PB@GO composite ensured the successful synthesis of rGO-wrapped FeS_2_ hollow nanocages (FeS_2_@rGO), resulting in stable potassium storage capacities (264 mAh g^−1^ at 50 mA g^−1^ for 50 cycles; 123 mAh g^−1^ at 500 mA g^−1^ for 420 cycles) [108]. The rGO network effectively buffered the volume change, established a conductive network for ion/electron transfer, and restrained the shuttle effect, thereby contributing to the excellent durability of the FeS_2_@rGO PIB anode. This strategy is also viable in the bimetallic metal chalcogenide-based nanocomposites with stronger synergism between individual phases. Since CNTs can interconnect the metal chalcogenide nanoparticles and form interlaced charge transfer highways, a composite with MnSe/FeSe_2_ nanoparticles interconnected by CNTs (denoted as Mn-Fe-Se/CNTs) was developed by solution-based reaction and subsequent in situ selenisation procedure (Fig. 13a) [110]. The CNT network prominently enhanced the potassium diffusion coefficient and hence led to the superiority in rate capability (83 mAh g^−1^ at 800 mA g^−1^) and cycling durability (141 mAh g^−1^ at 50 mA g^−1^ after 70 cycles) of Mn-Fe-Se/CNTs compared with Mn-Fe-Se. Similarly, another composite with NiS/FeS heterostructures in N-doped carbon interlaced with CNTs (Ni–Fe-S-CNT) was fabricated by coprecipitation and thermal sulphurisation [109], as displayed in Fig. 13b. Ascribed to the 3D hierarchically conductive network and the synergism of NiS/FeS heterostructures, the shortened ion diffusion aisles and the well-maintained structural integrity imparted Ni–Fe-S-CNT with superior potassium storage performance compared with NiFeS and FeS. With the same synthetic approach, N-doped carbon-coated CoSe_2_/NiSe_2_ heterostructures interconnected by CNTs (CoSe_2_/NiSe_2_@NC-CNTs) were designed, whose heterointerface facilitated the potassium storage performance [111]. According to the theoretical calculations, the CoSe_2_/NiSe_2_ heterointerface provided external K^+^ adsorption sites and dynamically facilitated the potassium storage reaction, thereby unveiling the preferable interfacial features of metal selenide-based heterostructures.

**Fig. 14:**
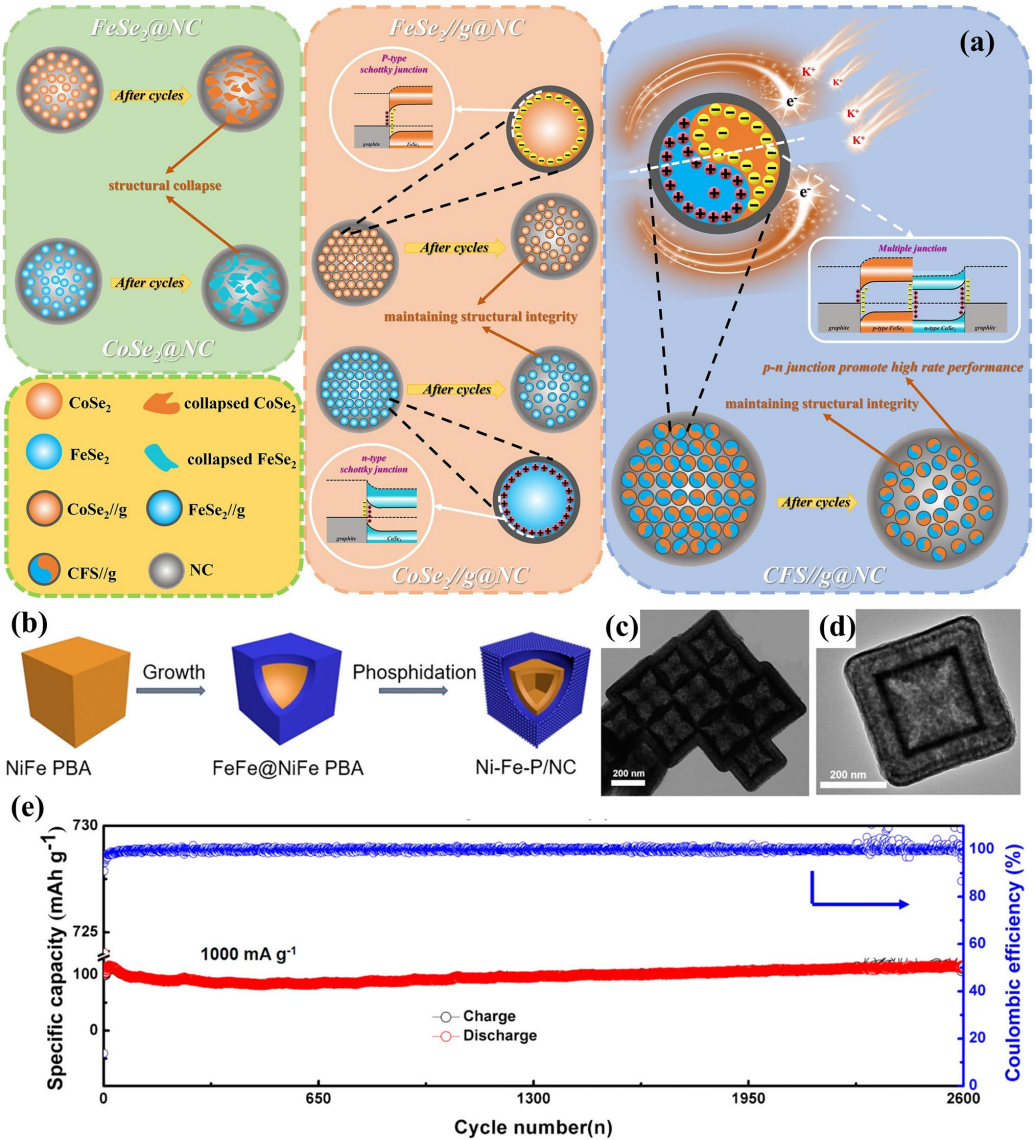
**a** Superior potassium storage property of CFS//g@NC. Reproduced with permission from Ref. [112], Copyright 2023, Elsevier. **b** Synthetic route, **c, d** TEM images, and **e** long-term cyclability at 1000 mA g^−1^ of Ni–Fe-P/NC. Reproduced with permission from Ref. [39], Copyright 2019, American Chemical Society

External carbon shells can serve as conductive layers and elastic buffers, which have been pursued to further reinforce the internal heterostructures. To alleviate the structural deformation of Ni_3_S_4_/Co_9_S_8_ heterostructures, a typical procedure comprising PDA coating of NiCo-PBA, in situ pyrolysis, and sulphurisation was employed to develop the N-doped carbon nanocube-confined Ni_3_S_4_/Co_9_S_8_ nanoparticles (abbreviated as NCS@NC) [156], as illustrated in Fig. 13c. The heterojunction in this composite anode accelerated ion/charge diffusion and the carbon shell maintained the primitive structure upon long-term cycling, which endowed the NCS@NC with an incredible lifespan (417.7 mAh g^−1^ at 2 A g^−1^ after 1000 rounds) as a PIB anode. In another work, the successful integration of Ni_3_S_2_-Co_9_S_8_ heterostructures with the open-ended hollow structure (developed by etching of the NiCo-PBA precursor), PDA-derived carbon layers, and rGO conductive substrate endued the resulting hierarchical composite (denoted as Ni-Co-S@rGO) PIB anode with negligible capacity degradation (0.089% per cycle at 10 A g^−1^) during long-term cycling [114]. Apart from the distinguished performance in PIB half cells, the Ni-Co-S@rGO||K_2_FeFe(CN)_6_ PIB full cell maintained a stable capacity of ~ 400 mAh g^−1^ at 2 A g^−1^ after 300 cycles with a steady operating voltage of ~ 1.68 V (Fig. 13d, e), thereby indicating the practicability of metal sulphide-based heterostructures in potassium storage due to the abundant voids, excellent mechanical robustness, and favourable heterointerfaces. This case suggests that self-standing electrodes can effectively resolve the particle agglomeration and ensure the energy density to satisfy practical PIBs. To systematically decipher the effect of heterojunction, a fabulous Janus heterostructure with graphite layer-coated CoSe_2_-FeSe_2_ in PDA-derived N-doped carbon (abbreviated as CFS//g@NC) was elaborately developed [112]. Excitingly, as portrayed in Fig. 14a, the dual heterojunctions (p-n and Schottky junction) and the graphite layer not only circumvented the structural fracturing and particle agglomeration but also expedited the ion/electron migration. It is worth noting that the existence of heterointerfaces triggered the charge accumulation phenomenon and hence caused the internal electric field, thereby promoting the potassium-ion diffusion and favouring the rate capability of CFS//g@NC. Hence, tremendous endeavours have supported the efficaciousness of heterostructures in advanced potassium storage from experimental and theoretical perspectives.

#### Metal Phosphides

Despite the dazzling theoretical capacity of metal phosphides, the pronounced volume expansion caused by the larger size of K^+^ and relatively low conductivity remain key challenges for their large-scale use in PIBs. Therefore, the development of hierarchical hollow architectures is the dominant approach to exalt the potassium storage properties of PBA-templated metal phosphide-based nanocomposites. As illuminated in Fig. 14b, the in situ growth of FeFe-PB on NiFe-PBA enabled the creation of the double-shelled composite with Ni–Fe-P encapsulated in N-doped carbon (nominated as Ni–Fe-P/NC) [39]. TEM images of Ni–Fe-P/NC reveal the double-shell cubic architecture with a conspicuous gap (~ 80 nm) between the outer and inner shells (Fig. 14c, d). This peculiar structural feature facilitated the electrolyte infiltration and mitigated the structural attenuation upon cycling, thereby resulting in considerable cycling durability (172.9 mAh g^−1^ at 0.5 A g^−1^ after 1600 cycles). When the current density was elevated to 1 A g^−1^, a reversible capacity of 115 mAh g^−1^ was maintained after 2600 cycles, as demonstrated in Fig. 14e. To enhance the mechanical strength, the 3D graphene skeleton was used to encapsulate PB-derived FeP hollow nanospheres (denoted as 3DG/FeP), during which the Ostwald ripening process played a part in inner void formation [113]. The combination of the 3D conductive substrate and hollow nanoparticles ensured the structural integrity and hence led to excellent capacity retention upon cycling (97.6% retention at 2 A g^−1^ after 2000 cycles). In the above cases, one advantageous factor is the Kirkendall effect during the thermolysis of PBAs that facilitates the formation of stress buffer hollow space without devastation of parent morphologies, which is beneficial to the long-term cycling lifespan.

#### Others

Aside from the conversion/alloying-type PIB anode materials, intercalation-type materials have also been deemed as the ideal commercial materials owing to their distinct voltage plateau. Notably, the utilisation of PBA templates facilitates the metal species (Zn, Fe, Co, Ni, etc.) decoration and increases the specific surface area, thereby providing exceptional potassium storage activities. Motivated by this, the N-doped graphitised carbon anode containing Co nanoparticles (abbreviated as Co-NC) with fast potassium storage kinetics was synthesised by the in situ carbonisation of ZnCo-PBA [62]. The strong Co–N bonds induced by the homogeneous dispersion of Co nanoparticles not only constructed a successive conductive network but also promoted K^+^ diffusion/adsorption, which enabled the fast and stable potassium storage behaviour of Co-NC.

Most studies related to PBA-templated nanocomposites concentrate on the design and modification of anode materials for PIBs, while few of them pay attention to the innovation of intercalation-type cathodes. For instance, K_0.6_CoO_2−*x*_N_*x*_ porous nanoframes were designed starting from CoCo-PBA by the sintering procedure with K_2_CO_3_ as the K source, where N substitution enlarged the interlayer spacing and amended the electronic configuration [81]. Consequently, this layered PIB cathode presented excellent cyclability (~ 80% capacity retention over 400 cycles at 50 mA g^−1^) and performed well when coupled with the graphite anode. Considering the efficaciousness of the N substitution tactic in layered materials, MnFe-PBA-derived P3-typed K_0.5_Mn_0.67_Fe_0.33_O_1.95_N_0.05_ (KMFON) nanosheets were fabricated using KOH as the K source and evaluated in PIB half cells and full cells [82]. The PBA-templated route enabled the N substitution and endowed KMFON with superior reversible capacities (104.2 mAh g^−1^ at 20 mA g^−1^ in the PIB half cell; 88.5 mAh g^−1^ at 20 mA g^−1^ in the PIB full cell with pitch-derived soft carbon as the anode) compared with the N-free counterpart. The suitable operating voltages and excellent energy densities of K-based metal oxides derived from PBAs make them feasible for practical PIB systems. Although experimental and theoretical findings manifest the feasibility of N doping for the acceleration of K^+^/electron transport in PIB layered cathodes due to the manipulation of atomic/electronic structures, there remains a bottleneck to enhance their reversible capacities (< 100 mAh g^−1^).

### Summary of Alkali-Ion Storage Applications

**Fig. 15:**
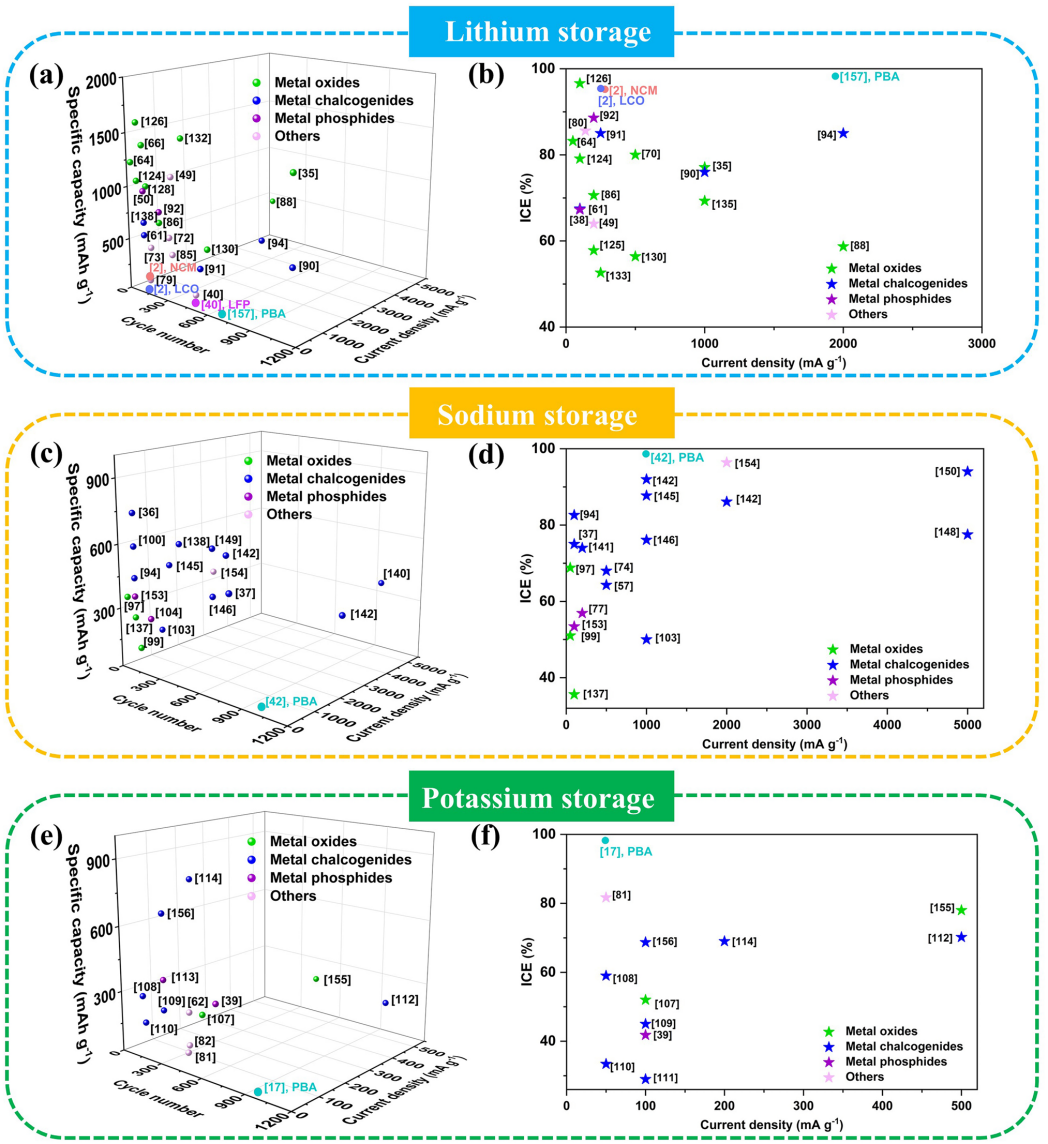
Comparison of cycle life and ICE values of PBA-templated nanocomposites and conventional materials in **a, b** lithium, **c, d** sodium, and **e, f** potassium storage

In light of the above, orchestrated endeavours have been dedicated to employing PBA-templated nanocomposites (metal oxides, metal chalcogenides, metal phosphides, etc.) as advanced electrode materials (anodes and cathodes) for AIBs (LIBs, SIBs, and PIBs). To directionally design AIB electrodes to adapt to different ion sizes of Li^+^, Na^+^, and K^+^, there are several principles to be followed: (1) precise selection of monomers and synthetic routes for desirable compositional and structural features of the resulting products; (2) abundant void space to buffer the volume fluctuation (conversion/alloying-type anodes, particularly) and allow electrolyte infiltration; (3) hierarchical architectures with strengthen structural robustness, affluent active centres, and enhanced energy density; (4) proper utilisation of endogenous and exogenous conductive matrices; (5) heterojunctions with preferable interfacial properties for boosted lithium/sodium/potassium kinetics. The further exploitation of PBA-templated materials can provide a platform for the advancement of AIB technologies. To identify the merits and demerits of various PBA-templated nanocomposites in alkali-ion storage, some key parameters including cycling durability and ICE values are evaluated in Fig. 15. PBA-templated metal oxides can perform well in lithium storage but can’t afford the larger size of Na^+^ and K^+^ ions. As for sodium and potassium storage, PBA-templated metal chalcogenides outperform their counterparts in reversible capacities and cycling durability due to their superior electrical conductivity. PBA-templated metal phosphides deliver satisfying reversible capacities but are subject to dissatisfactory electrical conductivity. Most alloying-type and conversion-type electrodes (especially metal oxides, metal chalcogenides, and metal phosphides) are subjected to low ICE values (< 80%) owing to the large alkali-ion consumption during the initial discharge process and the intensified side reactions of nanomaterials. Although their practical applications in AIBs are very infant, there have been many successful attempts to utilise alloying-type and conversion-type materials derived from PBAs to assemble AIB full cells, manifesting their promising practicability when rationally implementing compositional and structural optimisation with PBA templates. As for AIB cathodes, PBA-templated nanomaterials deliver prolonged cycle life and elevated capacities but present lower ICE values compared with conventional ones (e.g. NCM, LFP, and LCO) [2, 40]. Although PBAs deliver excellent stability and safety when investigated as AIB cathodes compared with PBA derivatives, most of them suffer from inferior reversible capacities (< 100 mAh g^−1^) and lower working voltages (< 3 V) that are insufficient for industrialisation in practical batteries [17, 42, 157]. Since ICEs are pivotal in practical applications, prelithiation/presodiation/prepotassiation techniques and the development of hierarchical composites are viable approaches. Additionally, when preparing a specific type of electrodes, nanocomposites derived from similar PBAs with same redox couples usually feature similar electrochemical behaviours. This can be a useful guideline for the directional design of PBA-templated AIB electrodes with desirable properties (operating voltages, reversible capacities, etc.).

**Fig. 16:**
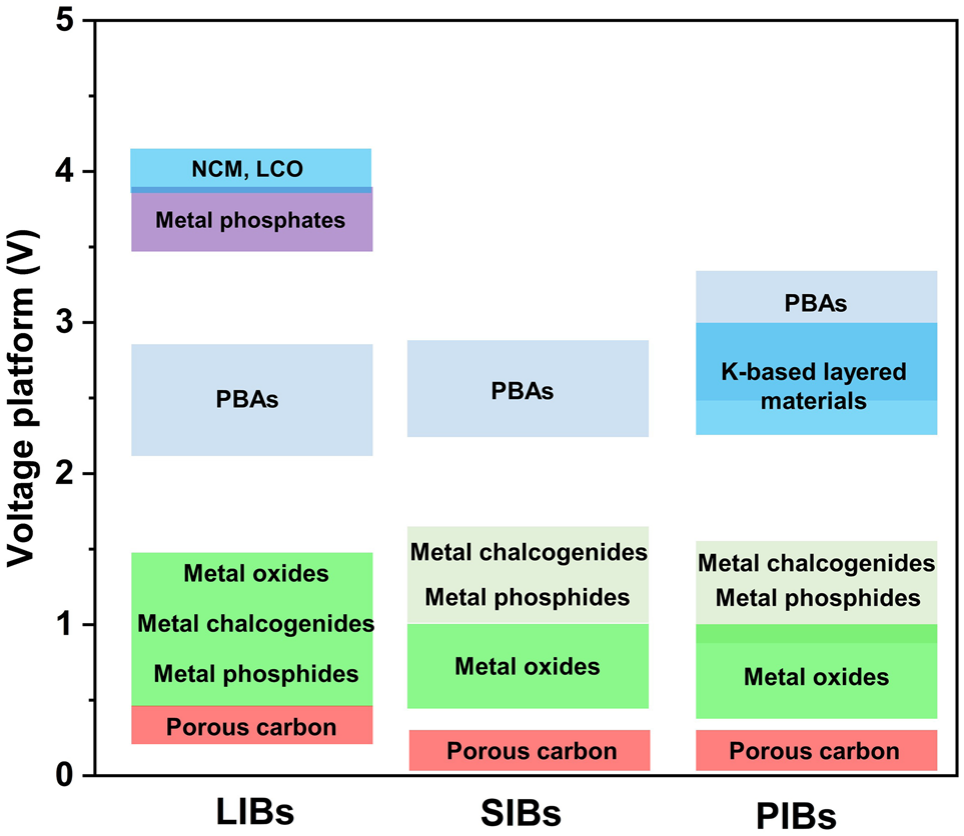
Working voltages of PBA-templated nanocomposites when investigated as LIB, SIB, and PIB electrodes

As for the practical aspect, there have been some attempts to assemble AIB full cells to reflect the practicability of PBA-templated materials, during which operating voltages and kinetics matching ought to be considered. Voltage plateaus and cutoff voltages of electrode materials during half cell measurements are vital parameters for their applications in AIB full cells because the overall energy density can be determined by both anodes and cathodes. To direct the matching of PBA-templated nanocomposites in AIB full cells, the voltage plateaus of various nanocomposites in lithium/sodium/potassium storage are summarised in Fig. 16. Apparently, materials with higher voltages can be suitable cathodes, while the ones with lower voltages can be suitable anodes. During the full cell assembly process, both voltages and N/P ratios are instrumental to the resulting performances and survivability in extreme conditions of AIB full cells [50]. AIB cathodes with proper interlayer spaces and higher electronic conductivity are conducive to ion intercalation/mobility and the inhibition of phase degradation, whose working voltages and capacities predominantly impact their full cell performance. For AIB anodes, researchers fancy developing materials with excellent safety performance, mitigated volume effect, and steady voltage platforms. Some research works reported the successful application of PBA-templated alloying-type and conversion-type electrodes in AIB full cells, but some of these materials fail to meet the commercial requirements due to the relatively high operating voltages and large-slope redox platforms. These drawbacks can be reconciled by selecting suitable cathode materials with higher working voltages and compositional integration for working voltage optimisation, thereby opening new avenues for the practicality of alloying-type and conversion-type materials. The future exploration of PBA-templated AIB electrodes is expected to keep pace with practical AIB systems.

## Conclusion and Perspective

The innovation of LIBs and the excavation of “beyond Li” batteries remain subjects of utmost significance, which provide unprecedented opportunities for PBA-templated nanocomposites with designable compositions and structures. These materials, with facile preparation, fabulous physicochemical properties, and tailorable electrochemical behaviours have been extensively investigated from experimental to theoretical studies rendering them as promising electrodes for LIBs/SIBs/PIBs. Despite the enormous progress of PBA-templated protocols in AIB electrode development, both advantages and challenges are present in their further advancement.

Despite the standard open framework structure of PBAs allowing the insertion/extraction of charge carrier alkali ions, the detrimental structural water and crystal vacancy-driven structural fragility are prone to cause subpar reversible capacities and dissatisfying cycling durability. In contrast, PBA-templated nanomaterials offer the following merits when employed as AIB electrodes: (1) retainable regular morphology with open porous texture, enlarged surface area, and abundant active sites; (2) phase homogeneity and excellent crystal orientation of active components; (3) vast defects and adequate void spaces induced by the Kirkendall effect for stress buffering; (4) hierarchical architectures with well-developed conductive networks ensuring high conductivity and balancing the advantages of nanoengineering and microengineering; (5) heterojunctions and/or heteroatoms modulating the electronic/atomic configuration for accelerated lithium/sodium/potassium diffusivity; (6) synergism of various electrochemically active components contributing to enhancing reversible capacities.

**Fig. 17:**
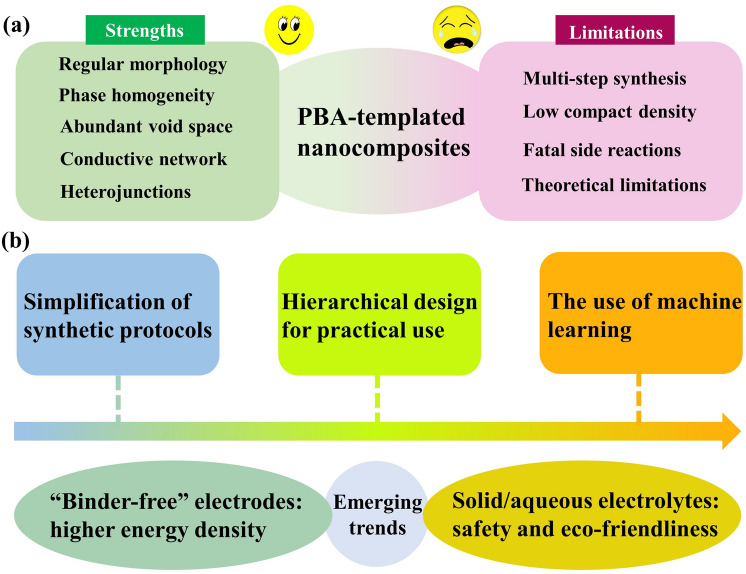
**a** Strengths/limitations and **b** developmental roadmap of PBA-templated nanocomposites

The prominent advantages of PBA-templated materials prompt the continuous pursuit of their applications in practice, but some intractable challenges hinder their mass production and commercialisation. To comprehensively evaluate PBA-templated nanocomposites, their strengths and limitations are summarised in Fig. 17a. Firstly, multi-step synthetic methods for these nanomaterials are too tedious for scale-up manufacturing compared with the conventional solid-state method, whose reproducibility and yields require further optimisation to meet industrial requirements. Therefore, from the economic perspective, the skilful selection of raw materials and the simplification of synthetic routes are emerging trends for the development of PBA-templated nanocomposites. Secondly, despite the amplified surface area and enhanced specific capacity, the inevitable shortcomings of PBA-templated nanomaterials including poor compact density and intensified surface side reactions severely taper their practicability. To tackle these issues, increasing attention has been devoted to PBA-derived hierarchical materials that manifest excellent electrolyte/electrode contact and synchronously warrant excellent volumetric energy density and high electrochemical efficiency. The SEM/TEM techniques are insufficient for microstructure analysis due to the overlap of nanocrystalline, so it is viable to apply state-of-the-art characterisations such as small-angle neutron scattering (SANS), small-angle X-ray scattering (SAXS), and dynamic light scattering particle size analysis (DLS) to statistically analyse these materials [158, 159]. Third, most related studies concentrate on electrochemical performance evaluation and experimental results but neglect theoretical and practical insights. Purposeless and reduplicative experimental trials are pernicious to the validity of as-designed PBA-templated materials, which require in-depth theoretical support to ensure the rationality of experimental schemes and avoid unnecessary energy consumption. Machine learning (ML) models can attain the goal of directional material design by establishing databases of structure–activity correlation and will play a critical role in the material science and energy industry in the future. With the rapid development of ML models, it can be assumed that material databases related to PBA-templated nanocomposites as advanced AIB electrodes will occur to direct targeted fabrication and application of these materials with the aid of the empirical link between their compositional/structural properties and electrochemical behaviours in AIBs. For a better understanding, the roadmap for the future development of PBA-templated nanocomposites is demonstrated in Fig. 17b.

Based on the advantages and challenges of PBA-templated nanocomposites, there are some potential developmental directions for these materials in next-generation AIBs. The retention of carbon skeletons is a prevailing methodology to construct elastic buffers and conductive matrices but may be prone to present lower volumetric/mass energy density. The mass and volume of electrochemically inert components are pivotal to the resulting energy density. Hence, the meticulous control of the carbon content will be a critical issue for PBA-templated carbonaceous materials, stimulated by which “binder-free” electrodes have become emerging candidates for AIBs. It is noteworthy that PBAs can be promising templates/precursors during the design of “binder-free” electrodes with the aid of robust conductive substrates, where the smaller volume and lighter weight of electrochemically inactive components are pivotal to the volumetric/mass energy density and hence play an important part in practical AIB systems. To attain this goal, PBAs can be controllably anchored on hard substrates and then pyrolysed under proper conditions, which is a prevailing strategy to develop “binder-free” electrodes with the PBA-templated route. Safety is a prerequisite for AIBs and can be realised by the conspicuous Li^+^/Na^+^/K^+^ insertion voltage platforms. Intercalation-type cathodes (i.e. LiCoO_2_ and LiFePO_4_) and anodes (i.e. graphite and hard carbon) with stable voltage plateaus will still occupy a predominant position in the future commercially available AIBs, whose reversible capacities can be further enhanced by integrating other components with additional alkali-ion storage capability. Meanwhile, their working voltages (carbon anodes particularly) need to be further optimised for the prevention of fatal dendrite growth leading to battery failure. The PBA-templated method is suitable for the elaborate design and modification of these intercalation-type electrodes from molecular and atomic levels. Another way to warrant the safety of practical batteries is to inhibit the uncontrollable alkali metal dendrite deposition on separators leading to the short circuit of batteries [160]. PBA-templated nanocomposites show excellent ability in the consumption of metal dendrite and hence are suitable for separator modification. Furthermore, the advent of novel electrolytes (i.e. solid-state electrolytes, aqueous electrolytes) is conducive to the adaptability to extreme conditions and the safety performance of AIB systems. Compared with conventional organic electrolytes, solid-state electrolytes show good processability and can result in higher energy density, while aqueous electrolytes display pronounced merits in safety and stability [161, 162]. The excellent chemical compatibility of PBA-templated nanocomposites makes them suitable battery components in various electrolyte systems. Pristine PBAs have been widely employed as electrodes for aqueous AIBs, but the applications of PBA derivatives in aqueous batteries are still limited [161, 163]. However, the successful case of metal oxides in aqueous SIBs indicates more possibilities for PBA derivatives as aqueous battery electrodes [139]. In light of the above, the most likely applications of PBA-templated nanocomposites in practical batteries are as follows: i) active components for “binder-free” electrodes; ii) modifiers for commercial AIB electrodes and separators; and iii) electrodes or electrolyte fillers in solid-state/aqueous AIBs. With the closer collaboration of experimentalists and industrialists and the improvement in fundamental understandings, it can be entailed that the gap between laboratory study and practical use of PBA-templated nanocomposites in AIBs will be eliminated. The strengthened connection among experimental attempts, theoretical investigations, and industrial reality will be the main driving force for this process.
